# Elongated TCR alpha chain CDR3 favors an altered CD4 cytokine profile

**DOI:** 10.1186/1741-7007-12-32

**Published:** 2014-05-09

**Authors:** Catherine Reynolds, Deborah Chong, Eleanor Raynsford, Kathryn Quigley, Deborah Kelly, Julia Llewellyn-Hughes, Daniel Altmann, Rosemary Boyton

**Affiliations:** 1 Lung Immunology Group, Infectious Diseases and Immunity, Department of Medicine, Imperial College, Hammersmith Hospital, Du Cane Road, London W12 0NN, UK; 2 Molecular Biology Laboratories, Natural History Museum, Cromwell Road, London SW7 5BD, UK

**Keywords:** CD4 T cell, T cell receptor, Cytokine, Transgenic, Mouse, Th17, Th1, Th2

## Abstract

**Background:**

CD4 T lymphocyte activation requires T cell receptor (TCR) engagement by peptide/MHC (major histocompatibility complex) (pMHC). The TCR complementarity-determining region 3 (CDR3) contains variable α and β loops critical for pMHC recognition. During any immune response, tuning of TCR usage through progressive clonal selection occurs. Th1 and Th2 cells operate at different avidities for activation and display distinct transcriptional programs, although polarization may be plastic, influenced by pathogens and cytokines. We therefore hypothesized that CDR3αβ sequence features may intrinsically influence CD4 phenotype during progression of a response.

**Results:**

We show that CD4 polarization involves distinct CDR3α usage: Th1 and Th17 cells favored short TCR CDR3α sequences of 12 and 11 amino acids, respectively, while Th2 cells favored elongated CDR3α loops of 14 amino acids, with lower predicted affinity. The dominant Th2- and Th1-derived TCRα sequences with14 amino acid CDR3 loops and 12 amino acid CDR3 loops, respectively, were expressed in TCR transgenics. The functional impact of these TCRα transgenes was assessed after *in vivo* priming with a peptide/adjuvant. The short, Th1-derived receptor transgenic T cell lines made IFNγ, but not IL-4, 5 or 13, while the elongated, Th2-derived receptor transgenic T cell lines made little or no IFNγ, but increased IL-4, 5 and 13 with progressive re-stimulations, mirrored by *GATA-3* up-regulation. T cells from primed Th2 TCRα transgenics selected dominant TCR Vβ expansions, allowing us to generate TCRαβ transgenics carrying the favored, Th2-derived receptor heterodimer. Primed T cells from TCRαβ transgenics made little or no IL-17 or IFNγ, but favored IL-9 after priming with Complete Freund’s adjuvant and IL-4, 5, 9, 10 and 13 after priming with incomplete Freund’s. In tetramer-binding studies, this transgenic receptor showed low binding avidity for pMHC and polarized T cell lines show TCR avidity for Th17 > Th1 > Th2. While transgenic expression of a Th2-derived, ‘elongated’ TCR-CDR3α and the TCRαβ pair, clearly generated a program shifted away from Th1 immunity and with low binding avidity, cytokine-skewing could be over-ridden by altering peptide challenge dose.

**Conclusion:**

We propose that selection from responding clones with distinctive TCRs on the basis of functional avidity can direct a preference away from Th1 effector responses, favoring Th2 cytokines.

## Background

In CD4 immunity, different contexts of antigen recognition, whether in the ‘natural setting’ of disease or the experimental setting of antigen-priming adjuvant regimens, favor the preferential development of effector populations that belong primarily to Th1, Th2 or Th17 subsets [1,2]. In diseases such as allergic asthma, there is strong, local Th2 immunity, while influenza infection induces a Th1 profile [3,4]. There are several mechanisms driving CD4 T cells into these alternate differentiation fates, including the local cytokine milieu. This partly reflects the experimental designs most accessible for reductionist models (that is, adding recombinant cytokines to purified, naïve cells to track polarization). However, it has relevance extending to the mechanisms involved in natural infection. Dendritic cell (DC) programming through IL-12 or IL-18 leading to Th1-polarization is commonly modeled by Toll-like receptor (TLR) agonism with bacterial products, the prototypic example being TLR agonism by bacterial lipopolysaccharide [5]. The nature of stimuli driving a Th2 environment is less clear. IL-10 and IL-4 are candidates for the promotion of an innate Th2-priming environment. However, IL-10 or IL-4 knockout DC can drive Th2 responses [6]. Clues as to the supply of a pro-Th2 DC program have come from looking at Th2-associated pathogen responses. Schistosome egg antigen (SEA), which induces a robust Th2 program in DC, capable even of overriding bacterial Th1 signals [7], depends on a relatively non-activated transcription profile in the DC, somewhat reminiscent of a tolerogenic profile [8]. This is in keeping with the view that activation of Th2 cells may encompass reduced T cell receptor (TCR) avidity and/or co-stimulation and altered signaling, synapse formation and off-rates.

Several lines of evidence suggest that Th2 activation has different avidity requirements for Th1 activation. A basic precept is that the TCR reads different potencies of activation to initiate different effector outcomes with the ‘strength of stimulation’ required for a Th1 response greater than for a Th2 response [9]. Furthermore, more sustained engagement of the TCR by pMHC is required to polarize TCR transgenic lymphocytes into a Th2 program [10]. The serial triggering model of T-cell activation offers an explanation for these observations; since any given pMHC complex must activate several receptors for cell triggering, the affinity of the interaction must be low and/or the off-rate fast to enable disengagement to occur and the more sustained the necessary interaction, the greater this will be the case [11]. There are many studies on the relative dose of antigen required for a Th1 or Th2 response; a common observation is that Th1 responses require relatively high concentrations, and Th2 responses much lower [12]. Possible mechanisms for a relationship between antigen dose and T cell polarization may be both through a direct effect on differential CD3 signaling [13] and through the ability to modulate expression of co-stimulatory molecules expressed by CD4 cells; high antigen dose can favor Th1 development through dose-dependent up-regulation of CD40L [14]. Indeed, it has been argued that Ca2^+^ signaling may be reduced in Th2 response and that the threshold interaction necessary for inducing Ca2^+^ signaling may not be reached by Th2 cells [15].

In several models, pMHC complexes showing reduced affinity interactions with TCR are associated with preferential skewing to a Th2 response. T cells specific for proteolipoprotein (PLP) 131-159 and selected under Th2-favoring conditions show a shift in peptide-TCR primary contact residues compared with Th1 clones [16].

While avidity maturation on a per-cell basis is a property of the B cell and not the T cell repertoire, T cell clonal selection leads to the progressive appearance of selective TCR usage [17-19]. For example, the repertoire of TCR response to sperm whale myoglobulin 110-124 in adjuvant is initially diverse, then progressively losing lower affinity clones and resulting in an oligoclonal population of TCRβ receptors of intermediate affinity [18]. Similarly, pigeon cytochrome c peptide specific TCRβ chains show strong selection for CDR3 length and residues characteristic of antigen binding, compatible with progressive clonal maturation and the suggestion of population-level affinity maturation [19]. Repertoire maturation in development of the CD8 response to *Listeria* involves narrowing of the TCR repertoire, associated with increased affinity [17].

Knowing that development of a response involves selective fine-tuning from available receptors and that avidity requirements differ for activation of different Th subsets, it would be expected that they might selectively expand different TCR repertoires. We previously showed that selection of favored TCRs from the peptide-specific pool differs under Th1 or Th2 conditions [20]. When an initial, mixed pool of primed cells was divided into Th1 or Th2 polarized cultures, different TCR sequences were preferentially selected under the two conditions. Across different pMHC combinations, there was no clear pattern or homogeneity evident in the selection for TCRβ usage, but Th2 conditions preferentially selected elongated CDR3α sequences. The example of the response to PLP 56-70/H-2A^g7^ was studied in detail. Screening of polarized Th line libraries suggested that while Th2 cultures favored these elongated CDR3α loops but also encompassed receptors with shorter loops, the long loops could never be found in Th1 cultures. Molecular modeling offered a potential explanation, predicting a bulky, obstructive interaction of reduced affinity for the elongated Th2 receptors. Thus, contrary to experiments with the DO11.10 TCR transgenic mouse, whereby a Th1-derived TCR can be skewed to mediate either Th1 or Th2 effector functions [21], in a physiological polarizing environment, receptor features may be preferentially selected so as to skew the future memory response for an appropriate cytokine profile [22]. While it had been envisaged that the cytokine program was faithfully transmitted to progeny cells by chromatin remodeling, polarization is now perceived as a plastic event [2,23-28]. In this context, there may be evolutionary advantage in building information on the appropriate response into the TCR itself, so that the response cannot be diverted to a pathogen-inappropriate response by the local inflammatory environment.

We here investigate this hypothesis by generating TCR transgenics carrying an elongated CDR3, Th2-derived TCRα chain. We show that cells carrying this receptor, particularly when paired with the appropriate TCRβ partner, facilitate cytokine skewing away from a Th1 program. This is the first time to our knowledge that a causal link between TCR repertoire maturation and Th effector polarization has been shown. The findings suggest that the program for recall of a context-appropriate phenotype may be influenced not just by heritable epigenetic changes, but also in the choice of dominant TCRs themselves.

## Results

### TCR usage and co-stimulatory molecule expression in polarized CD4 T cell lines

We previously described the relatively homogeneous TCRα usage associated with a polarized Th2 response to PLP 56 to 70 [20,22]. A noteworthy feature of this TCR usage was the choice of CDR3α loops that were elongated, an observation extended across several different pMHC combinations, suggesting that the Th2 cytokine environment had influenced preferential utilization of TCRs with these structural features. In order to establish the impact on cytokine polarization of Th2-derived receptors with these features, TCRα chain transgenic lines expressing a representative, elongated CDR3α receptor were generated. In order to select such a TCR sequence, we sequenced TCRα and β chains from polarized Th lines specific for this pMHC combination (H2-A^g7^/PLP 56 to 70). Primed lymph node cells (LNC) from mice primed with peptide in Complete Freund’s adjuvant (CFA) were split into cultures stimulated with antigen *in vitro* under polarizing Th1, Th2 or Th17 conditions. To generate Th1 lines, cells were cultured in medium containing IL-2, IL-12 and anti-IL-4. To generate Th2 lines, cells were cultured in medium containing IL-2, IL-4 and anti-IFNγ. For Th17 lines, cells were initially cultured in anti-IFNγ, anti-IL-4, IL-6 and TGFβ and expanded in medium containing IL-2 and IL-23. Each set of conditions yielded a repertoire of TCRs with reproducibly distinct features (Tables 1, 2, 3). In each case, the distinctive CDR3s were a characteristic of the TCRα and not the TCRβ repertoire. Under Th1 conditions, cultures predominantly favored a Vα15Jα10 (TRAV10D TRAJ58) receptor with a 12 amino acid CDR3α, AASREGTGSKLS. We never found this receptor or ones similar to it in cultures derived from the same LNC pool but polarized under Th2 or Th17 conditions. Cells cultured under Th2 polarizing conditions favored very different TCRα usage, a quarter of the sequences comprising a Vα9Jα42 (TRAV17 TRAJ50) receptor with an elongated, 14-amino acid CDR3α loop, ALEGIASSSFSKLV. This was not accompanied by any overt selection for dominant TCRβ chains. Interestingly, cells cultured under Th17 polarizing conditions were also distinctive, a large proportion of sequences (44%) being Vα11Jα21 (TRAV4D-3 TRAJ27) comprising a short, 11 amino acid CDR3α loop (AAANTNTGKLT). Again, there was no preferred, expanded TCRβ chain. The findings suggested selection from the available pool primarily on the basis of TCRα sequence and permissible pairing of dominant TCRα chains with multiple possible β chain partners. The Th17 cells were similar to Th1-polarized cultures in being limited to short CDR3α loops. There was no significant difference in TCRβ chain CDR3 length seen in Th1 (12.3 ± 0.2), Th2 (12.3 ± 0.2) and Th17 (12.7 ± 0.2) TCR sequences. However, TCRα chain CDR3 lengths were significantly longer in Th2 (12.2 ± 0.2) than in Th1 (11.4 ± 0.1) and Th17 (11.1 ± 0.1) TCR sequences (*P* <0.0009).

**Table 1:** TCR α and β chain repertoires of a Th1 polarized T cell line

		**CDR3 region**		**CDR3 length**	**%**
TCR alpha	**EDSAIYFC**	**AASREGTGSKLS**	**FGKG**	**12**	**34**
	SDSAKYFC	ALEGRGGRALI	FGTG	11	20
	EDSGTYFC	AALPGTGSNRLT	FGKG	12	18
	GDSAMYFC	AAKNSGTYQR	FGTG	10	8
	GDSAIYFC	SASMTNNNNRIF	FGDG	12	6
	EDSGTYFC	AADSNYQLI	WGSG	9	4
	EDSGTYFC	AAETNSAGNKLT	FGIG	12	2
	EDSGTYFC	AADSNHQLI	FGSG	9	2
	EDSGTYFC	AAEAANYNVLY	FGSG	11	2
	EDSAIYFC	AASKPNNRIF	FGDG	10	2
	SDSAVYFC	ALSALGTGNYKYV	FGAG	13	2
TCR beta	DDSATYFC	ASSQGPLSNERLF	FGHG	13	22
	NEMAVLFC	ASSRSGDQDTQY	FGPG	12	18
	EDSAVYLC	ASSRDWGDTQY	FGPG	11	14
	DDSATYFC	ASSQEMQGQDTQY	FGPG	13	6
	EDSAVYLC	ASSPWGVQDTQY	FGPG	12	4
	QDSAVYLC	ASSLAGQGARSQNTLY	FGAG	16	4
	SQTSLYFC	ASSPGSNERLF	FGHG	11	4
	KDSAVYLC	ASSLVGAEQF	FGPG	10	4
	DDSATYFC	ASSKAGTGEDTQY	FGPG	13	2
	DDSATYFC	ASSQQGDQDTQY	FGPG	12	2
	DDSATYFC	ASSQEGTGVQDTQY	FGPG	14	2
	DDSATYFC	ASSQEGTGGDEQY	FGPG	13	2
	DDSATYFC	ASSQEGLSSYEQY	FGPG	13	2
	DDSATYFC	ASSQEGLGNYEQY	FGPG	13	2
	DDSATYFC	ASSQEMQGDQDTQY	FGPG	14	2
	QDSAVYLC	ASSNQNYAEQF	FGPG	11	2
	QDSAVYLC	ASSSRDWGDEQY	FGPG	12	2
	EDSAVYFC	ASSQAGTDTQY	FGPG	11	2
	EDSAVYFC	ASSSPGGSYEQY	FGPG	12	2
	SQTSVYFC	ASGDSQGANQAPL	FGEG	13	2

T cell lines were maintained in culture for one round of re-stimulation under highly polarizing conditions before taking RNA for TCR sequence analysis. TCRα and β chain repertoire analysis of an H2-A^g7^/PLP 56-70 specific Th1, Th2 and Th17 cell lines shows expansion of distinct, dominant TCRα chains in the absence of a dominant β chain. The frequency (%) of each unique TCR sequence identified is shown. CDR3 length is defined as the number of amino acids between the invariant C residue and the F/W-G-X-G motif. TCRα and TCRβ CDR3 region lengths are shown in each table. Data are representative of 50 TCRα and 50 TCRβ sequences for each T cell line. The mean CDR3 length for the TCRα chain is 12.2 ± 0.2 (SE) for Th2 cultures, 11.4 ± 0.1 (SE) for Th1 cultures, and 11.1 ± 0.1 (SE) for Th17 cultures.

The most dominant TCR alpha sequence is shown in bold.

Mean CDR3α length 11.42 + 0.13 (SE) (n = 50).

Mean CDR3β length 12.30 + 0.17 (SE) (n = 50).

**Table 2:** TCRα and β chain repertoires of a Th2 polarized T cell line

		**CDR3 region**		**CDR3 length**	**%**
TCR alpha	**SDSAKYFC**	**ALEGIASSSFSKLV**	**FGQG**	**14**	**24**
	GDSAAYFC	AVRGTNAYKVI	FGKG	11	16
	SDSALYYC	ALSDANNYAQGLT	FGLG	13	14
	GDSAAYFC	AAGDTNTGKLT	FGDG	11	10
	EDSAIYFC	AASRGNMGYKLT	FGTG	12	8
	GDSAAYFC	AALNTNTGELT	FGDG	11	6
	SDSAVYYC	ALVRDTGYQNFY	FGKG	12	4
	SDSAVYYC	ALGEDTNAYKVI	FGKG	12	4
	SDSAVYYC	ALGFQGGRALI	FGTG	11	4
	GDSAMYFC	AAPPMNYNQGKLI	FGOG	13	4
	TDSGTYFC	AMERODNYAOGLT	FGLG	13	2
	EDSGTYFC	AADNRIF	FGDG	31	2
	SDSAVYYC	ALGDREGGRALI	FGTG	15	2
TCR beta	QDSAVYLC	ASSFQTGGAETLY	FGSG	13	6
	NEMAVFLC	ASSSPTGGWNAEQF	FGPG	14	6
	EDSAVYLC	ASSNYAEQF	FGPG	9	6
	DDSATYFC	ASSLGTGDAEQF	FGPG	12	6
	DDSATYFC	ASSLRDNGDTQY	FGPG	12	6
	DDSATYFC	ASSOEAGGVDTQY	FGKG	13	4
	NEMAVFLC	ASSPRTTSGNTLY	FGEG	13	4
	NEMAVFLC	ASSPPTGSPNERLF	FGHG	14	4
	EDRGLYLC	GARDLWGGKNTLY	FGAG	13	4
	DDSATYFC	ASSQEGLDSYEQY	FGPG	13	4
	NEMAVFLC	ASSIEDSGTEVF	FGKG	12	4
	QDSAVYLC	ASSLRGHTEVF	FGKG	11	2
	ODSAVYLC	ASSVRDWGDTQY	FGPG	12	2
	QDSAVYLC	ASSLRNTEVF	FGKG	10	2
	DDSATYFC	ASSQEGWGPYEQY	FGPG	13	2
	DDSATYFC	ASSQGLGNYAEQF	FGPG	13	2
	DDSATYFC	ASSQEGTGGYAEQF	FGPG	14	2
	DDSATYFC	ASSQDGTGGYAEQF	FGPG	14	2
	DDSATYFC	ASSQGIYEQY	FGPG	10	2
	DDSATYFC	ASSPANSDYT	FGSG	10	2
	DDSATYFC	ASSQDGTIODTQY	FGPG	13	2
	DDSATYFC	ASSPDWDTTGQLY	FGEG	13	2
	DDSATYFC	ASSQDRSSSAETLY	FGSG	14	2
	DDSATYFC	ASSQEGTGGKEQY	FGPG	13	2
	NEMAVFLC	ASSSPTGGWNAWQF	FGPG	14	2
	EDSAVYLC	ASSPRGLYAEQF	FGPG	12	2
	EDSAVYLC	ASSRDWGSEQY	FGPG	11	2
	EDSAVYLC	ASSPDWGDEQY	FGPG	11	2
	SQTAVYFC	ASSGRTTANTEVF	FGKG	13	2
	SQGRTLYC	TCSADGSYEQY	FGPG	11	2
	EYSAMYLC	ASSSGGFAETLY	FGSG	12	2
	EYSAMYLC	ASRDWGETLY	FGSG	10	2
	SHSGFYLC	AWSLWSGVANERLF	FGHG	14	2
	SQTSLYFC	ASSDFSTEVF	FGKG	10	2

The most dominant TCR alpha sequence is shown in bold.

Mean CDR3α length 12.24 ± 0.20 (SE) (n = 49).

Mean CDR3β length 12.25 ± 0.20 (SE) (n = 51).

**Table 3:** TCRα and β chain repertoires ot a Th17 polarized T cell line

		**CDR3 region**		**CDR3 length**	**%**
TCR alpha	**EDSGTYFC**	**AAANTNTGKLT**	**FGDG**	**11**	**44**
	EDSGTYFC	AAEDNNNNAPR	FGAG	11	16
	EDSGTYFC	AAMNYNQGKLI	FGQG	11	6
	EDSGTYFC	AAVDYNQGKLI	FGQG	11	4
	TDSGTYLC	AMDMNNNNAPR	FGAG	11	4
	EDSGTYFC	AAEAPSSGQKLV	FGQG	12	4
	GDSAVYFC	AVSVDNYAQGLT	FGLG	12	4
	EDSGTYFC	AAMNTNTGKLT	FGDG	11	2
	EDSGTYFC	AANNYNQGKLI	FGQG	11	2
	EDSGTYFC	AAEGNSGTYQR	FGTG	11	2
	EDSGTYFC	AAEDSGGNYKPT	FGKG	12	2
	EDSGTYFC	AAYNYAQGLT	FGLG	10	2
	EDSGTYFC	AAEADTNAYKVI	FGKG	12	2
	EDSGTYFC	AAGPHNNNAPR	FGAG	11	2
	TDSGTYLC	AMER6TNTGKIJT	FGDG	12	2
	SDSAVTPC	AARSDTNAYKVI	FGKG	12	2
TCR beta	EDSAVYLC	ASSSTGGAHYAEQF	FGPG	14	8
	QDSAVYLC	ASSLVGQGDTQY	FGPG	12	6
	DDSATYFC	ASSQDQISQNTLY	FGAG	13	6
	DDSATYFC	ASSQDLGTSNERLF	FGHG	14	6
	SQTSVYFC	ASGDSAGGNSPLY	FAAG	13	4
	SQTSVYFC	ASAWGENTLY	FGAG	10	4
	ODSAVYLC	ASSLDTGYTEVF	FGKG	12	2
	QDSAVYLC	ASSLGQGTEVF	FGKG	11	2
	QDSAVYLC	ASSLAPGQGDERLF	FGHG	14	2
	QDSAVYLC	ASSLDQTNERLF	FGHG	12	2
	ODSAVYLC	ASSLAGANTGQLY	FGEG	13	2
	QDSAVYLC	ASSLDAGQNYAEQF	FGPG	14	2
	QDSAVYLC	ASSPPDTYEQY	FGPG	11	2
	ODSAVYLC	ASSPQGYQDTQY	FGPG	12	2
	ODSAVYLC	ASSLDWGEGNTLGL	FGAG	14	2
	SQTSVYPC	ASGDGTGGRDE0P	FGPG	13	2
	SQTSVYFC	ASG6GTASNERLF	FGHG	13	2
	S0TSVYFC	ASGETANTEV	FGKG	10	2
	SQTSVYPC	ASSDAGTGRDTEVF	FGKG	14	2
	SQTSVYFC	ASSDAAGGFIAEQF	FGPG	14	2
	SQTSVYFC	ASSDAGVTGQLY	FGEG	12	2
	SQTSVYFC	ASSDGQNTLY	FGAG	10	2
	SQTSVYFC	AGSGDWGDEQY	FGPG	11	2
	SQTSVYFC	ASSAGQQDTQY	FGPG	11	2
	SQTSVYFC	ASSDAGTGRDTEVF	FGKG	14	2
	SQTSVYFC	ASSDEGTKPDTEVF	FGKG	14	2
	SQTSVYFC	ASSDDRVNERLF	FGHG	12	2
	SQTSVYFC	ASSPSGTGSYEQY	FGPG	13	2
	SQTSVYFC	ASSDDRVNERLF	FGHG	12	2
	DDSATYFC	ASSQEGTGGDEQY	FGPG	13	2
	DDSATYPC	ASSQEKGQGYAEQF	FGPG	14	2
	NQTSVYFC	ASSSPFNSYNSPLY	FAAG	14	2
	NQTSVYFC	ASSLRTGGGGTEVF	FGKG	14	2
	SHSGFYLC	AWSHNRGNSDYT	FGSG	12	2
	EYSAMYLC	ASSGPSTGRDTEVF	FGKG	14	2
	EYSAMYLC	ASSRGDWGNEQY	FGPG	12	2
	NEMAVFLC	ASSMGTYAEQF	FGPG	11	2
	EDSAVYLC	ASSSLGGRNYAEQF	FGPG	14	2
	EDSAVYLC	ASSLGLGAETLY	FGSG	12	2

The most dominant TCR alpha sequence is shown in bold.

Mean CDR3α length 11.14 ± 0.06 (SE) (n = 50).

Mean CDR3β length 12.70 ± 0.18 (SE) (n = 50).

**Figure 1:**
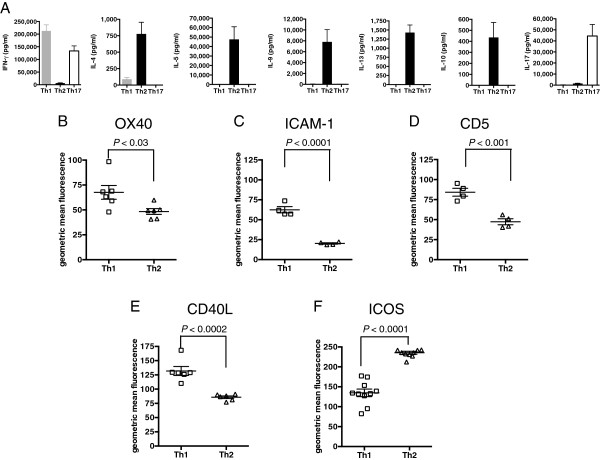
**Th1, Th2 and Th17 polarized T cell lines have distinct cytokine profiles and differ in expression of co-stimulatory molecules. (A)** Th1 (gray bar), Th2 (black bar) and Th17 (white bar) polarized cultures show the expected phenotype (note that, as described by others, Th17 lines can produce IFNγ). Data are shown for five independent Th1 (n = 5), Th2 (n = 5) and Th17 (n = 5) lines. This experiment was repeated on more than three separate occasions. **(B-F)** Highly polarized T cell lines derived from the same CFA/peptide immunizations were maintained in culture for one passage of re-stimulation (10 days) and then analyzed for expression of the indicated co-stimulatory molecules expressed on CD4-co-stained cells. This experiment was repeated on three separate occasions. Statistically significant differences are indicated (unpaired T test).

When assayed for release of IFNγ, IL-4, IL-5, IL-9, IL-13, IL-10 and IL-17, each of the polarized lines showed the expected profiles (Figure 1A). A caveat here is that, in line with observations from others using *in vitro* and *in vivo* models, T cells within Th17 cultures could flip relatively easily into co-expression of IL-17 and IFNγ [24-27] or single expression of IFNγ, (Additional file 1) particularly if derived from an *ex-vivo* inflammatory environment.

In line with the idea that selection from the initial pool under Th polarizing conditions acts through selection of clones with differing avidities, Th1 and Th2 lines developed with markedly differing profiles of co-stimulatory molecule expression (Figure 1B-F). Th1 lines show greater expression of CD40L, in line with the idea that CD40L-blockade preferentially blocks Th1 responses (Figure 1E) [14]. Expression of OX40, Intercellular adhesion molecule-1 (ICAM-1) and CD5 were also greater on Th1 lines (Figure 1B-D). Conversely, Th2 inducible T-cell co-stimulator (ICOS) expression was greater, as predicted by the finding that ICOS stimulation preferentially triggers Th2 cytokines (Figure 1F) [28]_._ The CD5 expression differences are reminiscent of observations in a recent paper by Mandl and colleagues, who showed that TCR transgenic CD5^hi^ clones accounted for high tetramer binding [29].

### Generation of TCRVα chain transgenics

**Figure 2:**
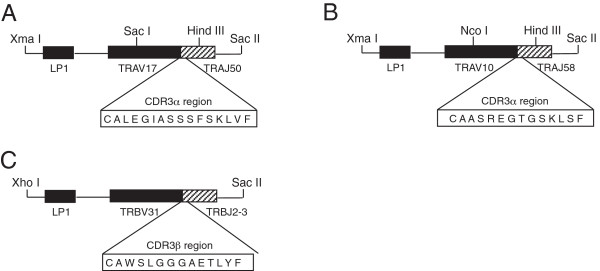
**Schematic diagram illustrating construction of the Th2 derived TCRα chain transgene with an elongated CDR3 region, Th1 TCRα chain transgene with a short CDR3 region, and Th2 TCRβ chain transgene. (A)** The Th2-derived TCRα chain transgene with an elongated CDR3 region, **(B)** Th1 TCRα chain transgene with a short CDR3 region, and **(C)** Th2 TCRβ chain transgene showing the recombined V and J gene segments and the amino acid sequence of the CDR3 regions. Intronic sequences flank the 3′ and 5′ ends of the coding regions and also separate the leader sequence (LP1) from the main coding region of the V gene. Transgenes were cloned into TCR α or β chain expression vectors that contain TCRα or β constant regions and endogenous promoter elements, using *Xma* I/*Sac* II sites for the TCRα and *Xho* I/*Sac* II sites for the TCR β transgene.

We expressed the immunodominant selected TCRα sequence (Vα9Jα42 (TRAV17 TRAJ50) receptor with an elongated, 14-amino acid CDR3α loop, ALEGIASSSFSKLV) in the pTα expression cassette, and made two independent transgenic founder lines expressing the dominant Th2-derived TCRα chain with an elongated CDR3 region (line 20 and line 34). We expressed the immunodominant selected TCRα sequence (Vα15Jα10 (TRAV10D TRAJ58) receptor with a 12-amino acid CDR3α, AASREGTGSKLS) in the pTα expression cassette, and made one transgenic founder line with the dominant Th1-derived TCRα chain with a shorter CDR3 region (line 30). All the transgenic lines were then backcrossed onto the original non-obese diabetic, H2-E transgenic (NOD.E) background in which the T cells had been characterized (Figure 2A, B).

### Transgenic expression of TCRVα chain with an elongated CDR3 does not favor Th1 responses in primed cells

With respect to TCR selection in Th2 T cell lines, the absence of any dominant TCRβ chain in cultures led us to hypothesize that transgenic expression of the TCRα chain with an elongated CDR3 region alone might be sufficient to impact on cytokine phenotype of the T cell response.

**Figure 3:**
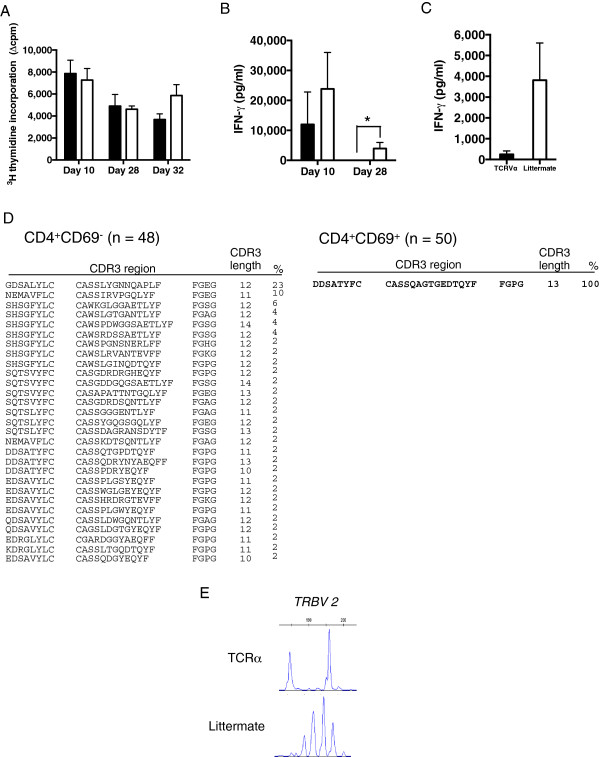
**The *ex-vivo *response of TCRα transgenic T cells show impaired IFNγ response and selection of a preferred TCRβ partner chain. (A)** TCRα chain transgenic and littermate controls were immunized with PLP 56 to 70/CFA on Day 0 and PLP 56 to 70/IFA on Day 28. T cell proliferation is similar in the two groups. Data shown are mean values ± SE (TCRα transgenic (black bars), n = 4 mice at Day 10, 6 at Day 28 and 7 at Day 32; littermate controls (white bars), n = 8 mice at Day 10, 8 at Day 28 and 9 at Day 32). The data are representative of three independently performed experiments. **(B)** Immediately *ex-vivo* at Day 10, both groups make similar amounts of IFNγ, but at Day 28 no IFNγ response is seen in the TCRα transgenics (*P <0.05; Mann-Whitney U test) or subsequently after re-challenge at Day 32, **(C)** TCRα transgenics show an impaired IFNγ response compared to controls. **(D)** T cells from the TCRα chain transgenics were harvested at Day 32, cultured with peptide for 48 h and TCRβ chain CDR3 analysis of CD4^+^ CD69^+^ (n = 50 sequenced receptors from cDNA of bulk, sorted T cell lines) and CD4^+^ CD69^−^ (n = 48 sequenced receptors from cDNA of bulk, sorted T cell lines) cells carried out. The TCRα chain transgenic impaired IFNγ phenotype correlates with clonal expansion of a dominant TCRβ chain (TRBV2, TRBJ2-5 CDR3 CASSQAGTGEDTQYF). **(E)** Spectratyping analysis of CD4^+^CD69^+^ TCRα chain transgenic and littermate control cells was carried out using V gene specific primers.

We initially assessed the functional impact of the elongated TCRα chain on CD4^+^ T cell responses by immunizing mice to look both at initial recall responses and subsequently after a second antigen boost *in vivo* (Figure 3). The nature of *ex-vivo* cytokine responses is heavily influenced by the adjuvants used for priming, with CFA giving strong Th1 polarization through the effect of *M. tuberculosis* on MyD88-dependent signaling [30]. We, therefore, assessed whether this TCR could confer propensity to develop a Th2 program in the face of a Th1-skewing, CFA priming regimen. Mice were immunized on Day 0 and Day 28 with PLP 56 to 70 in CFA and incomplete Freund’s adjuvant (IFA), respectively, and T cell responses sampled at days 10, 28 and 32. At each timepoint, T cell responses of TCRα chain transgenics were similar to littermate controls as judged by proliferation (Figure 3A). Interestingly, however, while the littermate controls generated a strong IFNγ response, this was absent after the initial recall in the TCRα transgenics at Day 28 (*P* <0.05) (Figure 3B). This suggested that use of this receptor was less effective at maintaining IFNγ transcription. When mice were boosted, we observed an IFNγ response in littermates but not in TCRα transgenics (Figure 3C).

T cells from the TCRα chain transgenics were harvested at Day 32, cultured with peptide for 48 h and CDR3 analysis of the CD4^+^CD69^+^ (n = 50) and CD4^+^CD69^−^ (n = 48) cells was carried out. The lack of an IFNγ response in TCRα transgenics after boosting correlated with selection of a single shared Vβ2 partner chain, as demonstrated by TCR sequencing and by spectratyping using V gene specific primers (Figure 3D, E). All the CD4^+^CD69^+^ cells expressed the same TCRβ chain, TRBV2 TRBJ2-5 with an elongated 13-amino acid CDR3 region ASSQAGTGEDTQY. Thus, the defective IFNγ recall response was associated with the clonal expansion of a population of cells expressing a single TRBV2 TRBJ2-5 chain with an elongated CDR3.

### Cytokine polarization evolves in cultured cell lines in line with favored TCRαβ selection

**Figure 4:**
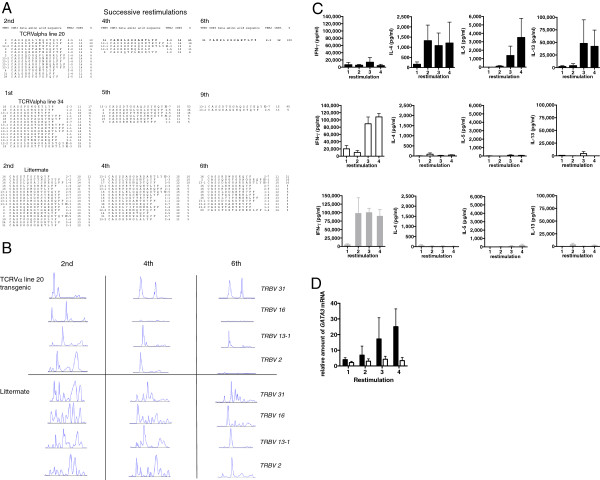
**Re-stimulation in non-polarizing conditions of TCRα transgenic cultures *in vitro *shows progressive focusing of favored TCRβ usage correlating with adoption of a Th2 cell phenotype.** CFA/peptide primed draining lymph nodes were used to establish T cell lines *in vitro* from TCRα transgenic lines 20 and 34 with an elongated Valpha CDR3 region, TCRα transgenic line 30 with a short Valpha CDR3 region, and littermate controls. Every 10 days, lines were re-stimulated with peptide and APC. **(A)** Analysis of the TCRβ chain repertoire of TCRα chain transgenic lines 20, 34 and littermate control T cell lines at successive re-stimulations. Sequence analysis of the repertoire of TCRβ CDR3 regions for TCRα chain transgenic and littermate control T cell lines at the re-stimulation numbers shown. The percent of each T cell line TCRβ repertoire attributed to each unique TCRβ chain sequence identified is shown. **(B)** cDNA from TCRα chain transgenic line 20 and littermate control T cell lines at re-stimulation numbers 2, 4 and 6 was used to spectratype Vβ gene families *TRBV2*, *TRBV13-1*, *TRBV16* and *TRBV31*. **(C)** IFNγ, IL-4, -5, -13 were measured at each re-stimulation of TCRα chain transgenic lines with an elongated CDR3 (line 20) (black bars) (n = 3), transgene negative littermate control lines (white bars) (n = 5) and TCRα chain transgenic lines with a short CDR3 (line 30) (gray bars) (n = 3). **(D)** Relative expression level of GATA-3 was determined by real-time PCR for TCRα chain transgenic lines with an elongated CDR3 (line 20) (black bars) (n = 3) compared to littermate control lines (n = 5). Error bars indicate SE. This experiment was repeated on three separate occasions. APC, antigen presenting cell.

In order to look in more detail at the relationship between progressive polarization and focusing of the TCR repertoire, we set out to look at polarization and TCRβ selection during progressive re-stimulation of T cell lines *in vitro*. This was done in the absence of exogenous polarizing factors in the medium. This allowed us to further explore the possibility that, with progressive re-stimulation and selection, there may be increasing focus on particular TCR pairs, leading to impaired Th1 responses and gradual promotion of Th2 responses. Primed draining LNC were used to establish T cell lines *in vitro*. Elongated CDR3 TCRα chain transgenic lines 20 and 34, short CDR3 TCRα chain transgenic line 30 and transgene negative littermate controls were primed with peptide in CFA and, 10 days later, LNC were harvested and T cell lines established. Line 20 and line 34 TCRα transgenic cells rapidly selected dominant TCRβ chain expansions (Figure 4A). By the fourth re-stimulation, over half of the TCRβ repertoires for the TCRVα transgenic lines comprised a single Vβ chain (although differing between the founder lines: VRBV31 TRBJ2-3; CAWSLGGGAETLYF in the case of line 20 and TRBV13-1 TRBJ2-7; CASSDTGGAQSSYEQY in line 34) and by the sixth re-stimulation, the TCRVβ repertoire was composed almost exclusively of this Vβ chain. These studies, along with the studies shown above in Figure 3, argue that several different TCRβ chains can pair with the elongated TCRα chain to yield the desired pMHC specificity and that in a given mouse or T cell line, one of several possible sequences may acquire clonal dominance. This selection of TCRβ chains in the context of pMHC activation was confirmed by spectratyping (Figure 4B). As early as the second re-stimulation, CD4 T cell lines from line 20 TCRα transgenics show constrained spectra, with a significant contraction of the TCRVβ chain repertoire for several Vβ gene families while spectra from littermate control T cell lines show TCRVβ diversity (Figure 4B).

We then looked at this TCR focusing during evolution of a given T cell line in relation to skewing of cytokine production. At the same time as making cDNA at each re-stimulation for TCR sequence analysis and spectratyping, cytokine production was analyzed by ELISA and RNA used for real-time analysis of subset-specific transcription factors. With progressive re-stimulations *in vitro*, the reciprocal nature of Th1 and Th2 polarization in littermate controls and TCRα transgenic lines with the shorter compared to those with elongated CDR3 regions is seen. TCRα transgenics with the elongated CDR3 region (line 20) make little or no IFNγ, but, after a lag of one re-stimulation, make large amounts of IL-4, IL-5 and IL-13 (Figure 4C). TCRα transgenic lines with the shorter CDR3 region (line 30) and non-transgenic controls make IFNγ, but no IL-4, IL-5 and IL-13. In line with this, *GATA-3* transcription is progressively up-regulated in TCRα elongated CDR3 transgenic cultures compared with littermate controls (Figure 4D).

There is no bias of the TCRVβ chain repertoire in naïve TCRVα chain transgenic splenocytes at Day 0 (Additional file 2A), or in a primary response in draining lymph nodes at Day 10 post-immunization as demonstrated by spectratype analysis (Additional file 2B).

### Generation of TCRαβ transgenics

The likely interpretation of the simultaneous appearance of dominant TCRβ chain sequences in the TCRα chain transgenic lines, adoption of a spontaneous Th2 phenotype and impaired Th1 program, was thus that features of this preferred TCRαβ pair were incompatible with effective maintenance of Th1 activation and, therefore, favored emergence of a Th2 response. We therefore expressed one of the immunodominant, selected TCRβ sequences (TRBV31 TRBJ2-3; CAWSLGGGAETLYF) in the pTβ expression cassette (Figure 2C), generated TCRβ transgenics, and crossed these to line 20 TCRα transgenics on the same NOD.E background.

### TCRαβ transgene impacts on *ex-vivo* T cell phenotype

**Figure 5:**
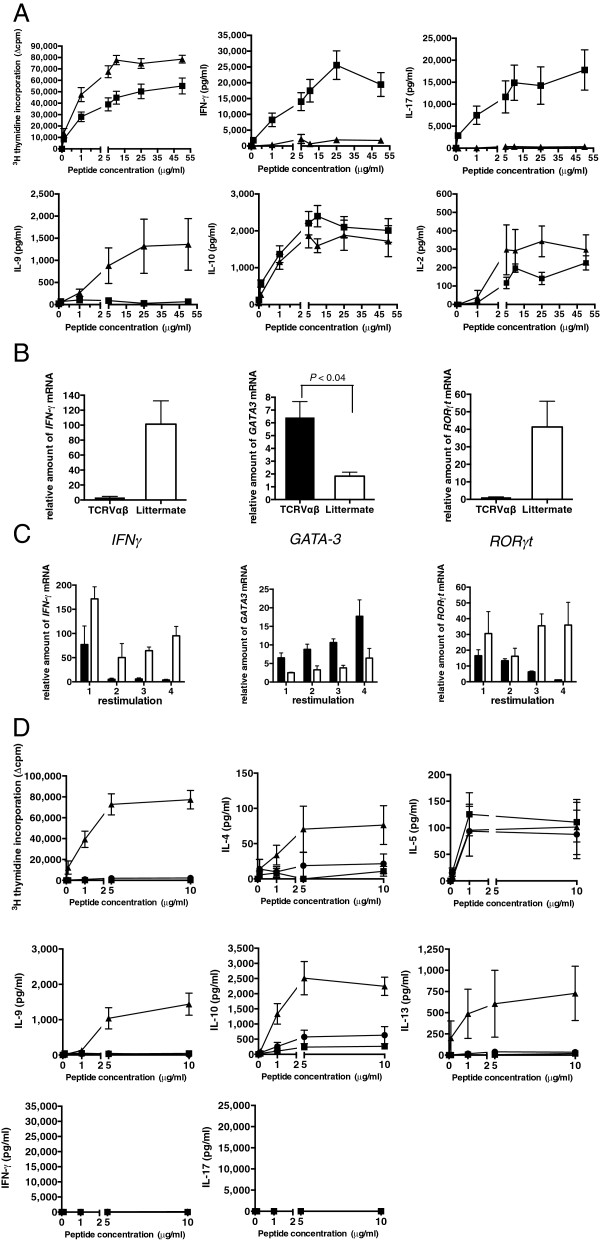
**TCRVαβ transgene pair impacts on cytokine production and transcriptional profile. (A)** TCRαβ transgenics or littermate controls were primed with peptide in CFA and DLN cells harvested at Day 10. ^3^H-thymidine incorporation and cytokine production was determined from TCRαβ transgenics (filled triangles; n = 6) and controls (filled squares; n = 10). Error bars indicate ± SE. This is representative of three separate experiments. **(B)** TCRαβ transgenic cell lines (black bars) (n = 3) and littermate control lines (white bars) (n = 5) were established from primed DLN cells from mice primed 10 days earlier with PLP56 to 70/CFA and re-stimulated every 10 days through to four cycles in the absence of any exogenous polarization. **(C)** At each re-stimulation the relative expression of *IFNγ*, *GATA-3* and *RORγT* was determined. Error bars indicate SE. **(D)** TCRαβ (filled triangles; n = 10), TCRα chain transgenics (filled circles; n = 12), and littermate controls (filled squares; n = 27) were primed with peptide/IFA and at Day 10, DLN cells were harvested and ^3^H-thymidine incorporation and cytokine production analyzed. These data are representative of three independently performed experiments. CFA, Complete Freund’s adjuvant; DLN, draining lymph nodes.

To assess the functional impact of the TCRαβ on primary *ex-vivo* T cell responses to peptide, TCRαβ transgenics and littermate controls were primed with peptide/CFA, again supplying a maximal Th1-skewing environment. At Day 10, T cell responses were analyzed *ex-vivo* and T cell lines established. While TCRαβ transgenics, as would be expected, show enhanced T cell proliferation responses to peptide, IFNγ and IL-17 responses were absent, and they made substantial amounts of IL-9 (Figure 5A). As before, immediately *ex-vivo* we could detect no IL-4, IL-5 or IL13 (data not shown). The absent production of IFNγ and IL-17 and enhanced production of IL-9 observed in the TCRαβ transgenic line was seen in the context of priming with the Th1 promoting adjuvant CFA making this result all the more striking. IL-10 production was not significantly different between the groups.

The phenotype of the TCRαβ transgenic lines was further confirmed by real time PCR for *IFNγ*, *GATA-3* and *RORγt*. The TCRαβ transgenic lines expressed high levels of *GATA-3* (*P* <0.004; unpaired t-test) and low levels of *IFNγ* and *RORγt* relative to the littermate controls in keeping with a Th2 phenotype (Figure 5B). Furthermore, *GATA-3* expression increased with successive re-stimulations, while *RORγt* expression decreased in keeping with the reduction in IL-17 production (Figure 5C). The level of *T-bet* transcription, however, showed no obvious pattern through re-stimulations of the TCR transgenic or WT lines (Additional file 3). This is in line with an increasingly complex model for T-bet in the Th1 program: in several models, Th2 polarization occurs in the face of T-bet transcription [31,32].

Because our investigations had used CFA priming, it was important to gauge the effect of immunizing TCRαβ double transgenic, TCRα chain single transgenic and littermate control transgenics with peptide in IFA lacking Th1-skewing killed mycobacteria (with the caveat that this is a less effective way of priming T cell responses). At Day 10, LNC were harvested and ^3^H-thymidine incorporation and cytokine production determined *ex-vivo*. The results are in line with the larger pMHC-specific T cell response in the TCRαβ transgenics, but confirm the Th2 bias: TCRαβ transgenic cells respond strongly to peptide making IL-4, IL-5, IL-9, IL-10 and IL-13, but no IFNγ or IL-17 (Figure 5D). TCRα chain transgenics, and littermate controls made IL-5 and small amounts of IL-10 only (Figure 5D). This is in keeping with the TCRαβ receptor supporting a Th2 phenotype.

### Addressing alternative hypotheses for cytokine-skewing in the TCR transgenics

The TCRαβ transgenic lines make more IL-2 than littermate controls, indicating T cell survival potential (Figure 5A). The high IL-2 response in the TCRαβ transgenic line, taken together with the high IL-9 and IL-10 response, implies that there is no global failure of T cell activation/survival *per se*, but merely a cytokine-deviated response. This appears to argue against a possible alternate hypothesis, that increased precursor number may have caused greater competition for IFNγ signals and decreased T cell fitness and/or memory [33-35]. To further address this issue, we immunized TCRαβ transgenics and littermate controls on Day 0 and Day 28 with PLP 56 to 70 in CFA and IFA, respectively, and looked at markers of cell activation and cell survival by real-time PCR and fluorescence activated cell sorter (FACS). Real-time PCR analysis of B-cell lymphoma-extra large (*Bclxl*) expression gave no indication of an enhanced propensity to apoptosis (Additional file 4A). Anti-apoptotic proteins, such as Bclxl, are involved in protecting mitochondrial integrity, for example, in the context of limiting growth factors [36]. Bclxl is induced on T cell activation and enhanced by co-stimulation of CD28 [37], and it is generally considered that over-expression of B-cell lymphoma 2 (Bcl-2) or Bclxl in T cells prevents death by neglect [33,38,39]. Furthermore, data from Gett and colleagues indicate that induction of Bclxl on sustained activation depends on stimulation strength and is associated with resistance to apoptosis, Bclxl increase being greatest in T cells that had received prolonged stimulation. If TCR transgenic cells were incapable of fully functional stimulation and destined for some form of sub-optimal stimulation or death by neglect, one would predict diminished activation of Bclxl in the transgenic response. In fact, we found no difference in *Bclxl* expression. Taken together with the other evidence shown here, including the strong IL-2 response on activation, we interpret the findings to mean that there is no deficit in activation of the response through the transgenic receptor, but rather, deviation of the transcriptional response to an altered cytokine profile. Furthermore, phenotypic analysis of the primed lymphoid populations at Day 28 showed no difference in activation/fitness as indicated by staining for CD127 (Additional file 4B). It has been shown by Whitmire and colleagues in a transgenic lymphochoriomeningitis virus (LCMV) response cell transfer model that only when the number of transferred T cell precursors was low did they generate fully functional memory cells that were CD127/IL-7R (high), with full capacity to produce cytokine and proliferate. Testing therefore whether it was possible that an excess of responder cells here correlated with a response phenotype that was in some way impaired, we analyzed T cell expression of CD127, and found that transgenic cells had enhanced, not reduced expression. Taken together with the other evidence presented here, this makes it unlikely that this was a model of sub-optimal activation. Furthermore, expression of CD62L was similar in the transgenic and littermate responder populations (Additional file 4C). Thus, the indication from IL-2 release, as well as apoptosis and activation markers is that the phenotype observed here constitutes an actively skewed functional preference, rather than a failure to trigger properly by the transgenic receptors.

It was hypothetically possible that injection of cognate peptide into a single chain TCRα and TCRαβ transgenic mice had triggered a cytokine storm due to the large number of responding T cells, and this had in turn caused thymic apoptosis leading randomly to a skewed cytokine outcome in surviving cells. To address this we conducted additional studies. ‘Cytokine storm’ is a term derived from the systemic response in toxic or septic shock [40]. Here, an acute, excessive spike of systemic pro-inflammatory cytokines leads to downstream events, often including large-scale lymphocyte apoptosis. In our studies the positive control stimulus of SEB led to the predicted spike in systemic pro-inflammatory cytokines, such as IFNγ and TNFα at 2 h, but no systemic change in cytokines was detected after injection of peptide/CFA (Additional file 5A, B). It was nevertheless possible that even in the absence of a systemic response there may have been some acute, storm-driven change in thymic populations accounting in a non-specific way for cytokine skewing. While SEB had a dramatic effect on overall thymocyte numbers and CD4/CD8 ratio in littermate control as well as TCRα and TCRαβ transgenics, no such effect was seen with cognate peptide (Additional file 5B). None of the peptide-primed non-transgenic controls showed preferred TCRβ chains in sorted CD4 SP cells from thymocytes (Additional file 5C). However, in one out of four of the mice analyzed, TCRβ chains in sorted CD4 SP cells from thymocytes in peptide-primed TCRα transgenics show preferential selection of a preferred TCR heterodimer, 44% of TCRs using a common TCRβ chain (Additional file 5D). On the basis of these experiments, the Occam’s razor hypothesis, that TCRα chains of differing structures and avidities can influence cytokine program, appears more likely than cytokine preference emerging as a stochastic event in the aftermath of a cytokine storm.

### T cell lines with TCRαβ receptor have low binding avidity

**Figure 6:**
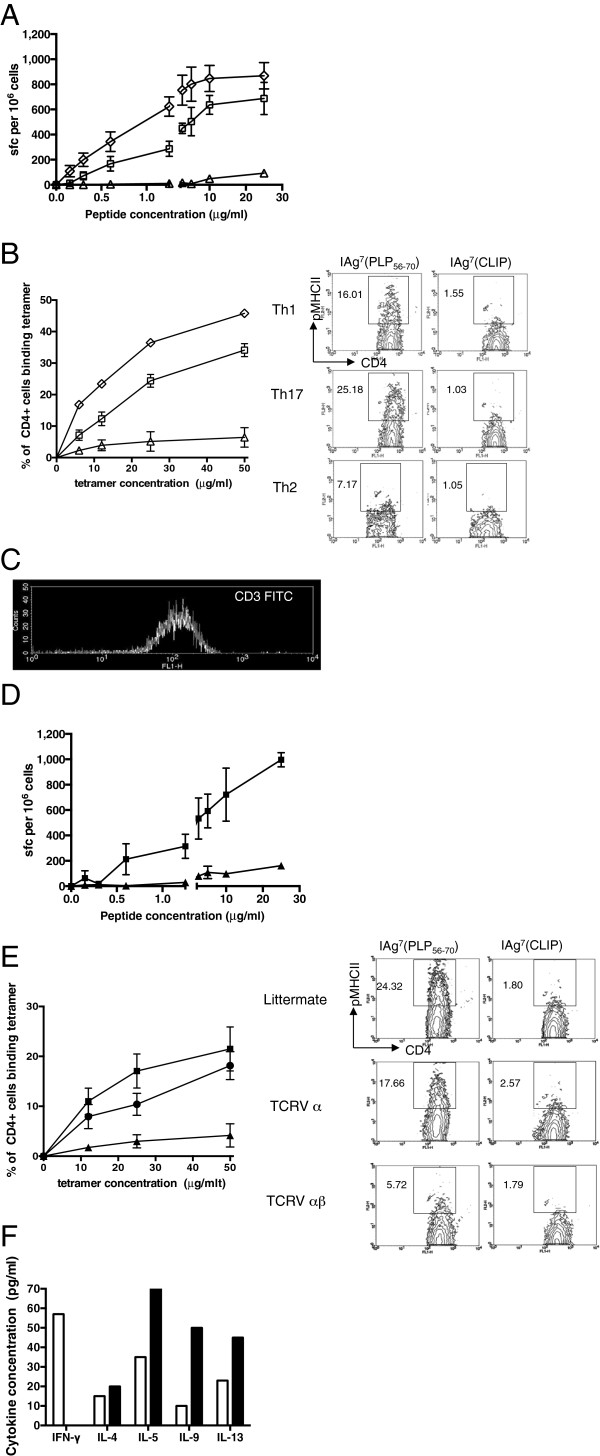
**Th2 derived TCRαβ has low avidity. (A)** ELISPOT assays for Th1 (open squares; n = 4), Th2 (open triangles; n = 3) and Th17 (open diamonds; n = 6) polarized, non-transgenic cell lines cultured for eight days detect IFNγ, IL-4 and IL-17 producing cells, respectively, in response to increasing concentration of PLP 56-70 peptide. **(B)** Th1 (open squares; n = 6), Th2 (open triangles; n = 6) or Th17 (open diamonds; n = 2) polarized, non-transgenic cell lines incubated with H2-A^g7^/PLP 56-70 or control (H2-A^g7^/CLIP 103-117) tetramers on Day 8 after primed draining lymph nodes (DLN) were incubated with peptide. Percentages shown are the difference between staining with H2-A^g7^/PLP 56-70 and control (H2-A^g7^/CLIP 103-117) tetramers. Data are representative of three separate experiments. **(C)** Th1 and Th2 polarized cell lines **(A, B)** have similar levels of CD3 expression. **(D)** ELISPOT assays for unpolarized, Th2 cytokine producing TCRαβ lines (filled triangles; n = 3) and IFNγ producing littermate control lines (filled squares; n = 5) cultured in the absence of polarizing cytokines through two successive 10-day cycles of re-stimulation with peptide, detect IL-4 and IFN-γ producing cells, respectively, in response to increasing concentration of PLP 56-70 peptide. **(E)** Unpolarized Th2 cytokine producing TCRαβ lines (filled triangles; n = 6), TCRα lines (filled circles; n = 6) and IFNγ producing littermate control lines (filled squares; n = 5) cultured through two successive 10-day cycles of re-stimulation with peptide, incubated with H2-A^g7^/PLP 56-70 or control (H2-A^g7^/CLIP 103-117) tetramers at the concentrations shown. Percentages shown are the difference between staining with H2-A^g7^/PLP 56-70 and control (H2-A^g7^/CLIP 103-117) tetramers. **(F)** Functional binding of tetramers determined by cytokine production from unpolarized TCRαβ lines and littermate controls following incubation with plate bound H2-A^g7^/PLP 56-70 tetramer. Cytokine concentration shown is a total minus background cytokine production for cells incubated with phosphate-buffered saline (PBS) only. The TCRαβ transgenic line (black bars) does not make IFNγ while the littermate control line (white bars) does. An IFNγ absent cytokine profile is elicited from the TCRαβ transgenic line.

We previously predicted from molecular modeling studies that the elongated CDR3 region of the TCRα chain, when paired with a preferred TCRβ chain, might result in a lower avidity interaction between the TCR and its pMHC ligand [20,22]. We therefore started by reappraising functional avidity in a peptide titration of Th1, Th2 and Th17 polarized, non-transgenic T cell lines cultured for eight days in polarizing medium and looking at IFNγ, IL-4 and IL-17 ELISPOTs, respectively. In these short-term lines, to achieve a response of 100 SFC/10^6^ cells requires about 3 times the peptide concentration in the Th1 lines compared to Th17, and about 250 times the peptide concentration in the Th2 lines compared to Th17 (Figure 6A).

We next looked at tetramer binding characteristics of Th1, Th2 and Th17 polarized, non-transgenic T cell lines cultured for eight days in polarizing medium and prepared from the same initial pool of primed LNC, using H2-A^g7^ tetramers loaded with either PLP56 to 70 or an irrelevant H2-A^g7^-binding peptide (CLIP103 to 117, PVSKMRMATPLLMQA). At all concentrations of tetramer tested, a significantly greater proportion of Th1 and Th17 polarized cells bound tetramer compared to Th2 cells despite equivalent levels of cell surface CD3 (Figure 6B, C). Similarly, in a peptide titration to examine the functional avidity of short-term T cell lines from TCRαβ transgenics relative to littermates, an equivalent number of IL-4 spot-forming cells in the TCRαβ transgenics require approximately 50 times the peptide concentration required for the IFNγ response in littermates (Figure 6D). We then compared tetramer binding characteristics of T cell lines derived from the Th2-type TCRαβ transgenic cells, TCRα chain transgenics with an elongated CDR3 and Th1-type cells from non-transgenic littermate controls. As tetramer concentration increased, a higher frequency of Th1 cells from the control littermate bound tetramer. However, no tetramer binding was detectable for the TCRαβ transgenic cells, indicating that the avidity of the interaction with the TCRαβ cells was too low for detection (Figure 6E). The TCRα chain transgenics with an elongated CDR3 had an intermediate binding avidity. We demonstrated a functional interaction between the tetramer and TCRαβ transgenic T cells by measuring cytokine production from transgenic cells cultured in the presence of plate-bound tetramer. In keeping with the Th2 phenotype, IL-4, IL-5, IL-9 and IL-13 but not IFNγ were detected (Figure 6F).

**Figure 7:**
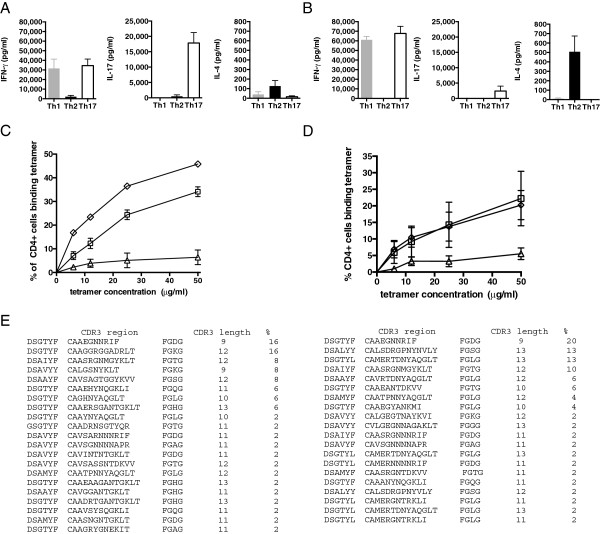
**Th17 polarized T cell lines have shorter TCRVαCDR3 length and higher tetramer binding avidity than Th1 and Th2 polarized cell lines.** Highly polarized Th1 (n = 6), Th17 (n = 6) and Th2 (n = 6) T cell lines derived from the same peptide immunizations of NOD.E mice were maintained in culture through four re-stimulations *in vitro*. Cells were analyzed for cytokine production and H2-A^g7^/PLP 56-70 tetramer binding avidity. At the first re-stimulation (Day 10), IL-17 production and high avidity tetramer binding by the Th17 cell lines are noted **(A, C)**. At the fourth re-stimulation (Day 40), IL-17 production and tetramer binding avidity of the Th17 cell lines have decreased **(B, D)** and the cytokine profile and tetramer binding characteristics now resemble those of the Th1 cell lines. **(E)** The TCRVαCDR3 chain repertoire of Th17 cells at the first (left hand panel) and fourth (right hand panel) re-stimulations were analyzed. The frequency (%) of each unique TCR sequence identified is shown. The mean TCRVαCDR3 lengths for the first and fourth re-stimulations are 10.95 ± 0.22 (SE) (n = 37) and 11.39 ± 0.21 (SE) (n = 51), respectively. The difference in mean CDR3α length between the first and fourth re-stimulation is statistically significant (*P* = 0.038) and shows progression to a TCR repertoire with longer CDR3α regions as cells lose their IL-17 producing phenotype and become more Th1 like in their tetramer binding characteristics and cytokine production.

The Th17 polarized T cell lines produced less IL-17 with each successive re-stimulation such that by the fourth re-stimulation cytokine production was more in keeping with that of a Th1 cell line (Figure 7A, B). We hypothesized that this shift in cytokine production would be accompanied by a reduction in tetramer binding avidity and longer TCRVαCDR3 length as T cells with a IFNγ producing phenotype clonally expand. In keeping with this, we observed a reduction in tetramer binding avidity between the first and fourth re-stimulations of polarized Th17 lines such that at the fourth re-stimulation, tetramer binding curves for Th1 and Th17 lines overlapped (Figure 7C, D). TCR repertoire analysis at the first and fourth re-stimulations confirmed that the change in cytokine production and tetramer binding avidity occurred alongside an increase in average TCRVαCDR3 length (Figure 7E). The mean TCRVαCDR3 lengths for the first and fourth re-stimulations are 10.95 + 0.22 (SE) (n = 37) and 11.39 + 0.21 (SE) (n = 51), respectively, (*P* = 0.038). The difference in mean CDR3α length between the first and fourth re-stimulation shows progression to a TCR repertoire with longer CDR3α regions as cells lose their IL-17 producing phenotype and become more Th1-like in their tetramer binding characteristics and cytokine production.

**Figure 8:**
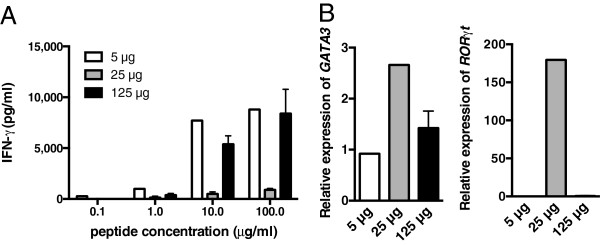
**Impact of different *in vivo *peptide priming doses on *ex-vivo *cytokine program.** Th2αβ TCR transgenic mice were primed in one hind footpad with 5 μg/ml (open bars), 25 μg/ml (gray bars) or 125 μg/ml (black bars) PLP 56-70 peptide in CFA. At Day 10 after immunization, DLN cells were re-stimulated with peptide at 0.1, 1.0, 10 or 100 μg/ml as indicated on the x-axis and assayed in triplicate cultures for IFNγ production by ELISA **(A)**. RNA was prepared from primed DLN immediately *ex-vivo* for real-time PCR analysis of *GATA-3* and *RORγt* transcription **(B)**.

In light of previous observations in TCR transgenics, notably the early studies by Hosken and colleagues [41] using the DO11.10 TCR transgenic line, it might be predicted that the Th2 TCR transgenic-skewed polarization may be overcome by altering peptide dose. Certainly, among the many factors that can skew cytokine bias would be peptide dose itself. In the DO11.10 TCR transgenic studies, it was shown that under otherwise equivalent *in vitro* primary culture of naïve cells, mid-range peptide doses favored the generation of moderate IFNγ responses, while either higher or lower doses favored a switch to development of more Th2-like responses, giving the IFNγ response curve the form of a bell-shaped curve [41]. We, therefore, performed a set of experiments in our system to examine the behavior of the Th2-derived transgenic TCR across a checker-board of low to high peptide priming dose and a low to high *in vitro* re-stimulation dose. Our previous experiments had typically utilized a priming and re-stimulation dose of 25 μg/ml, corresponding to 13 μM towards the upper-end of the concentration range that had been tested by Hosken and colleagues using naïve T cells *in vitro*. We initially primed Th2 TCRαβ transgenic mice with peptide at 5, 25 or 125 μg (Figure 8). Popliteal LNC were then challenged *in vitro* with a titrated dose of peptide from 0.1 to 100 μg/ml. As we had seen before, peptide priming with 25 μg/ml did not trigger an *ex-vivo* IFNγ response, though IFNγ could be elicited at either a higher or lower dose of peptide priming, 5 or 125 μg/ml. Thus, these findings are reminiscent of the studies by Hosken and colleagues, except that the mid-range peak of IFNγ production that had been seen by them for the DO11.10 TCR is here exchanged, using a Th2 TCR, for a low/high range peak of IFNγ production. Our curve is the reciprocal of the DO11.10 finding. Furthermore, the mid-range dose of priming with 25 μg/ml peptide was associated with increased transcription of both *GATA-3* and *RORγT*. Thus, while there is a constraint on IFNγ activation, this is peptide dose dependent and can be over-ridden by either high or low dose peptide priming.

**Figure 9:**
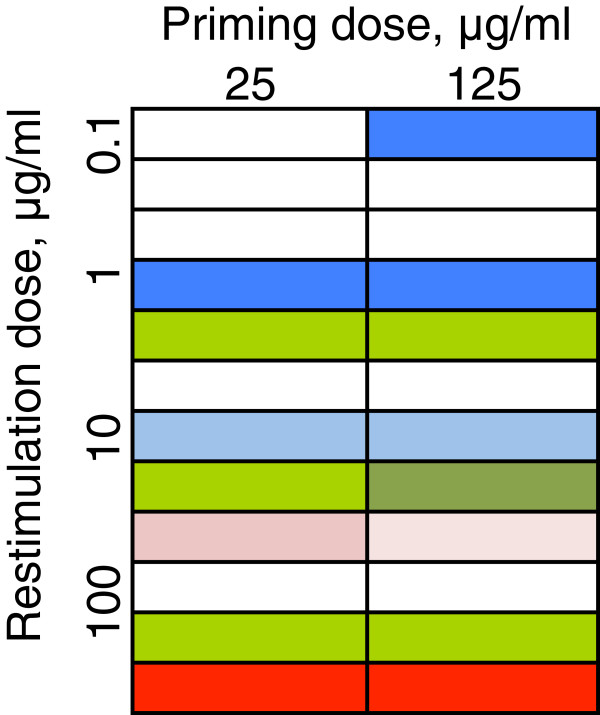
**Impact of different *in vitro *peptide restimulation doses on cytokine program of T cell lines.** Th2αβ TCR transgenic mice were primed with either 25 μg or 125 μg PLP 56-70 peptide in CFA and T cell lines were cultured from DLN cells for three cycles of re-stimulation and expansion using the peptide re-stimulation doses indicated. Triplet bars for each set of culture conditions are arranged in the order IL-4 (blues), IL-13 (greens) and IFNγ (reds). The darker the color, the greater the response for that cytokine. An unfilled (white) cell indicates no detectable response for that cytokine. For IL-4: unfilled, below detection limit; pale blue, response of <50 pg/ml; dark blue, response of >50 pg/ml. For IL-13, unfilled, below detection limit; green, response of <500 pg/ml; dark green, response of >500 pg/ml. For IFNγ (assayed by qPCR), unfilled (white) indicates relative expression of 1; pale pink indicates relative expression <25; dark pink indicates relative expression between 25 and150; red indicates relative expression >150.

**Figure 10:**
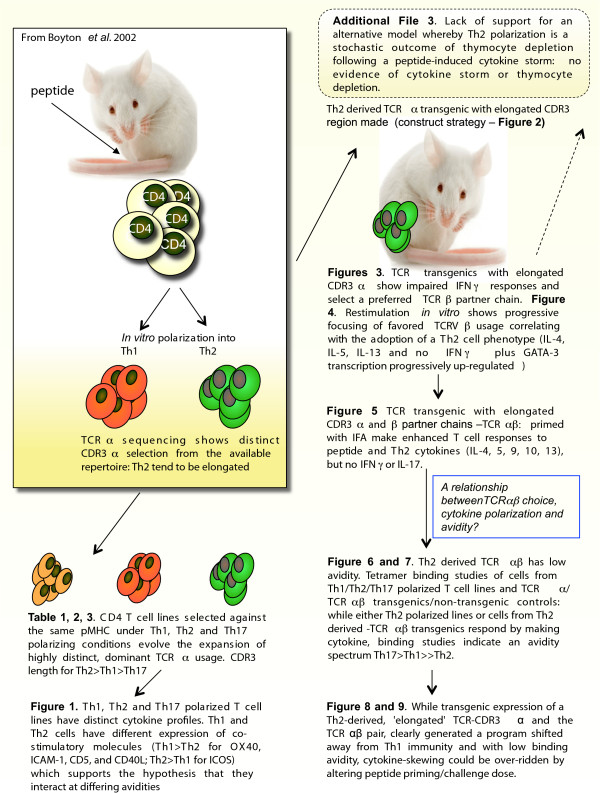
Schematic illustrating the flow of experiments used here to test the hypothesis that elongated TCR alpha chain complementarity-determining region 3 favors a Th2-skewed CD4 phenotype.

We then used this checker-board titration to analyze cytokine secretion in T cell lines from peptide primed Th2 TCRαβ transgenic mice, assaying their profile after the third re-stimulation *in vitro*. The findings are summarized in Figure 9. IL-4 responses are depicted in blue, IL-13 in green and IFNγ in red. The key arbiter of cytokine profile *in vitro* appeared to be the dose of peptide used for *in vitro* re-stimulation of the lines. That is, whether initial priming was with 25 or 125 μg, the T cells lines produced relatively large amounts of IL-4 and IL-13 but little or no IFNγ when re-stimulated with peptide in the range 0.1 to 10 μg/ml. However, irrespective of the initial priming dose, the T cell lines showed an IFNγ response when re-stimulated *in vitro* with 100 μg/ml peptide.Figure 10 is a schematic summarizing the flow of experiments designed here to test the hypothesis that elongated TCR alpha chain complementarity-determining region 3 favors a Th2-skewed CD4 phenotype.

## Discussion

There has been considerable effort devoted to elucidating the structural biology of the TCR complex, with 10s of structures solved, yet clear models for the way in which the ligand/receptor interaction between pMHC and TCR may give rise to the very diverse array of T cell effector programs have been elusive [42,43].

Most TCR transgenic lines are generated without regard for specific features of the cytokine profile of the parental cell from which they were derived. Experiments in some autoimmune models bear on this insofar as there are examples in which T cell clones were implicated in an autoimmune effector mechanism and the TCR transgenics derived from them can mediate a spontaneous disease phenotype dependent on preferential use of a similar cytokine profile [44]. Candon and colleagues generated transgenics for the TCR of a self-reactive Th2 clone, showing that a proportion of the mice spontaneously developed autoimmune gastritis characterized by eosinophilic infiltration of the gastric mucosa and Th2 differentiation of transgenic T cells in the gastric lymph node [45]. Appropriate Th polarization is most readily attributed to T cells engaging pMHC in the context of an antigen presenting cell (APC) that has been activated by innate signals to provide a particular, polarizing environment [46]. Could another aspect of the polarization reside in the TCR sequence itself? This would have evolutionary value as a contributory mechanism in polarization since, without it, the appropriateness, or otherwise, of the effector response could be at the mercy of influence by any other concurrent processes and/or infections. It is self-evident that there may be TCR structures that are ‘vanilla’ in nature and capable of being diverted readily to either a Th1 or Th2 program; DO11.10 is the prototypic example [21]. Knowing that TCRs of CD4 cells have been reported across a moderately broad range of affinities and also that affinity/avidity are key determinants of cytokine polarization, it may be predicted that TCRs of different structures would, all else being equal, show inherent bias to different effector profiles. The principle that TCR sequence correlates with function is accepted in the special case of the Treg versus Teff TCR repertoires [47], although even in this setting, phenotypic differences have proved difficult to define [48].

We show, using new TCR transgenic models, that an elongated TCRα derived from a strongly Th2-skewed T cell line retains imprinting of that effector program to the extent that, even in the face of an overwhelming Th1-polarizing environment, such as immunization in CFA containing *M. tuberculosis*, T cells can proliferate while lacking IFNγ responsiveness. While the polarization is mediated through the selection of appropriate CDR3α regions and the constraints on TCRβ selection are less apparent, the full Th2 program is clearly dependent on selecting the correct TCRαβ pair.

A starting point for the studies here was to reappraise more broadly our earlier observations about preferential selection of the TCR repertoire. This can be thought of as ‘avidity-associated functional maturation’ of the response; while it has long been known from many pMHC examples that the TCR repertoire can focus in on sometimes highly dominant receptor usage, we add the observation that this choice is highly influenced by factors driving Th1/Th2 polarization. A key observation, reiterated in the current study, had been that Th2 polarization is associated with CDR3α chains with somewhat elongated loops. In the present study, we have extended this analysis to investigation of Th17-polarized cultures. Once again, TCRβ chains remained relatively heterogeneous while TCRα sequences rapidly attain focused, preferential usage; although selected out of the same starting pool, the Th17 receptor usage is dissimilar to either Th1 or Th2 lines. Like Th1 lines, TCRα chains are invariably of the Th1-like, short, CDR3α type. We found no examples of elongated CDR3α chains in Th17 cells. This may be taken to mean that, like Th1 cells, Th17 activation is dependent on ‘high-end’ affinity activation and depends on activation that is incompatible with low affinity TCR activation. This is in line with the relatively easy plasticity from Th17 to Th1 lineages, although it is also known that there are differences between activation signals for Th1 and Th17 activation, such as dependence on PKCθ [49]. On the other hand, we found little similarity between the actual CDR3α sequences selected in Th17 and Th1 cultures. To our knowledge this study constitutes the first report of preferential TCR usage associated with the Th17 phenotype with the associated implications for differential avidity of Th17 cells. Th17 cells are predicted to be at the upper end of the avidity spectrum, with associated implications for differential signaling and synapse formation.

A number of studies have sought to relate changes in the TCR repertoire to the development of the T cell response, although not in the specific context of cytokine programs. Studies of CD8 responses have variously shown either a stochastic relationship between the initial response and the expanded repertoire, or substantial focusing with preferential selection of particular clonotypes, sometimes associated with enhanced avidity [50]. Those examples of CD4 repertoire selection that have been investigated in detail have also tended to focus on the progressive loss of low-affinity clones to achieve the ‘best fit’ [51,52]. What is unclear is how this model could be reconciled with a continuing requirement for differential avidities associated with alternate cytokine programs. Certainly, cells within a Th2 memory population can make IFNγ if reactivated in the context of innate Th1 signals [53]. That is, it might not be possible to maintain an overall dominance of Th2 clones in the face of a TCR repertoire progressively shifting to higher avidity. For example, in Th1/Th17 dependent experimental autoimmune encephalomyelitis (EAE) TCR transgenic models, strength of pMHC-TCR interaction is correlated with more aggressive, spontaneous disease [54]. Conversely, decreased CD4 expression by polarized Th2 cells has been shown to contribute to reduced TCR-induced phosphorylation and Ca2+ signaling [55].

Profiling of co-stimulatory molecules on Th1 and Th2 lines showed differences compatible with the notion of enhanced avidity promoting Th1 differentiation. Up-regulation of CD40L expression in Th1 cells is a previously well-documented example of this phenomenon, confirmed here [14]. Up-regulation of ICOS was a strong marker of Th2 differentiation, while CD5 up-regulation was a strong marker of Th1 differentiation. In line with the notion of CD5 up-regulation as a marker of enhanced avidity in Th1 cells, CD5 expression in thymocyte selection is correlated with avidity/signal intensity of the positively selecting TCR-pMHC interaction [56].

For differential engagement between pMHC on the APC and the TCR to lead to these very divergent transcription programs, there must be substantive differences in signaling, from initial formation of the immune synapse. A number of studies have previously considered differences in immune synapse formation and TCR signaling between Th1 and Th2 cells: most of these are compatible with the notion that the synapse leading to Th2 differentiation comprises a less tightly focused, lower avidity complex. Th2, but not Th1 cells, fail to cluster TCR at the cell-cell interface due to increased expression of cytotoxic T-lymphocyte antigen 4 (CTLA-4) [57]. In line with this, co-clustering of TCR with CD4 in lipid rafts is more common in Th1 cells [58]. Th2 immune synapses are morphologically distinct, characterized by the failure to exclude CD45 and ICAM-1 from the central zone [59]. These changes are presumed to contribute to diminished strength of signaling. Furthermore, a number of studies have considered mechanisms by which these differences might in turn lead to qualitative signaling differences and alternate transcriptional choices. Strength of signaling has been shown to affect the balance of NFATp and NFATc binding activity, thus directly regulating IL-4 transcription [60]. We predict from our TCR analysis of Th1, Th2 and Th17 lines that Th17 synapse formation and signaling would be distinct from the low avidity interactions characterizing Th2 activation and more reminiscent of Th1 activation.

Jameson and Masopust [61] have summarized a number of studies on the cost to the quality of memory of too much stimulation with the term, ‘everything in moderation: better memory by avoiding over-exertion.’ Is it possible that our Th2 TCRαβ transgenic model in some way elicits a response that is sub-optimal through excessive or inappropriate stimulation? While our observations here have focused largely on cytokine polarization of the initial response, all of the markers assessed here, including IL-2 activation, CD127 expression and anti-apoptotic markers indicate that responder cells in this transgenic system are fully fit to respond, they merely choose to do it in a different way.

The key evidence that cytokine program can be influenced by the selected TCRαβ sequence itself came with the observation that by transgenic expression of a Th2 derived receptor, we produced mice that are ‘Th1-averse’, even in the face of the most powerful possible stimulus to reverse this; despite showing strong proliferation in response to peptide, as would be expected, considerably higher than wild-type mice, there is no IFNγ or IL-17 response. Rather, the TCR transgenic mice make a strong Th2 response, not seen at all in the littermate controls. Why should this be the case? We speculate that it is an evolutionary failsafe to ensure pathogen-appropriateness of the response. In experimental immunology, we tend to work with reductionist systems where we can examine polarized Th1, Th2 or Th17 responses, unimpeded by other influences. However, in the natural host response to infection there will be many competing influences; thus, Th2 responses have presumably evolved to provide protection against parasitic infestation, yet in many parts of the world facing a major parasite burden, there is also a high level of infection with *M. tuberculosis*, itself providing a concomitant drive to Th1 and Th17 immunity [62]. Indeed, we have previously described another clinical example of this type in respect to the co-existence in the lung of sarcoidosis, which is associated with strong Th1 responses, and *Cryptococcus neoformans*, for which Th2 immunity is critical [63]. In complex settings, being able to avoid potentially dangerous reprogramming from pathogen-driven, cytokine milieu and promoting an appropriate effector response, is vital.

Cells with lower affinity TCRs adopt a Th2 phenotype when primed in the absence of competition from cells with higher affinity receptors [64]. Evidence that the Th2-derived TCR used in these studies are of low affinity comes from molecular modeling studies done in collaboration with Yvonne Jones’ laboratory [20]. In the present study, we were able to confirm those predictions using direct tetramer binding studies. We show here that while WT littermate primed cells respond to plate-bound tetramer by making IFNγ, TCRαβ Tg cells respond by making Th2 cytokines, but no IFNγ. This further emphasizes the fact that the property is purely one of the interaction between pMHC and TCR, uninfluenced by other contributory differences of co-stimulatory molecules. However, despite the fact that both cell populations can be activated by tetramers, binding to the transgenic T cells is sufficiently low as to be undetectable. The tetramer binding studies, taken in conjunction with the preferential TCR selection described by us in polarized lines, predict that the preference for TCR avidity will be Th17 > Th1 > Th2.

## Conclusion

We propose an additional and previously undefined mechanism for ensuring the cytokine appropriateness of CD4 immunity. TCR preferentially selected under Th2 conditions tended to use elongated CDR3α loops and when expressed in transgenics, skewed responses away from secretion of IFNγ, favoring Th2 cytokines. Focusing on the T cell repertoire in antigen specific adaptive immunity can bring with it information not just about pMHC specificity, but also about the qualitative nature of the appropriate cytokine response.

## Methods

### Ethics statement

The work described in this manuscript was covered by a Home Office Project License and approved by the Imperial College Ethical Review Process.

### Mice

To generate mice expressing an anti-PLP56-70/H2-Ag^7^ TCRα chain with the elongated CDR3α region, rearranged TRAV17J50 segments amplified from NOD.E genomic DNA and cDNA derived from a Th2 NOD.E T cell line against PLP56-70 and containing the CDR3 region (CALEGIASSSFSKLVF) were subcloned into pTalphaCass [65] (Figure 2A). Mice expressing a Th1 NOD.E T cell line derived anti-PLP56-70/H2-Ag^7^ TCRα chain (TRAV10/J58) with a shorter CDR3 region (CAASREGTGSKLSF) were also generated (Figure 2B). To generate mice expressing the anti-PLP56-70/H2-Ag^7^ TCRβ chain that pairs with the Th2 line-derived TCRα chain, rearranged TCRBV31J2-3 (CAWSLGGGAETLYF) segments amplified from NOD.E genomic DNA were cloned into pTbetaCass (Figure 2C). (C57BL/6xCBA) F_2_ oocytes were microinjected and TCR-positive founders identified by PCR and Southern. Two TCRα chain transgenic lines with elongated CDR3 regions (called TCRAV17J50elongatedCDR3-line 20 and TCRAV17J50elongatedCDR3-line 34) and one TCRα chain transgenic line with a short CDR3 region (called TRAV10J58shortCDR3-line30) are described here. All transgenic lines underwent at least five generations of backcrossing to NOD.E mice [66]. One TCRβ chain transgenic line is described here in detail, and termed the TCRBV31J2-3 line. The TCRβ transgenic line was crossed with the TCRα transgenic line 20 to make a TCRαβ transgenic line expressing the elongated TCRαCDR3 and its partner TCRβCDR3 backcrossed onto a NOD.E background.

### Immunization and T cell proliferation assays

PLP56 to 70, DYEYLINVIHAFQYV, was used for priming T cell responses and the substituted analog carrying lysine for tyrosine at positions 57 and 59 for *in vitro* re-stimulation of cells [67] (Biosynthesis Inc, Lewisville, Texas, USA). The substituted analog is necessary for *in vitro* studies as the original sequence is poorly soluble and cytotoxic. The altered peptide retains the ability when used *in vivo* to trigger the expected EAE phenotype [67]. Mice were immunized with 25 μg peptide in CFA or IFA (Sigma-Aldrich, Gillingham, Dorset, UK) in the footpad or flank. At Day 10 (unless otherwise stated) draining lymph nodes (DLN) and spleen were removed and cell suspensions were prepared in HL-1 medium (Lonza, Basel, Switzerland). Cells were cultured in triplicate in 96-well plates in the presence of peptide for three days. [^3^H]Thymidine was added 18 h before termination, and cultures were harvested (MACH III M Harvester 96) for beta scintillation counting (Wallac 1450 Microbeta TRILUX).

### T cell lines

T cell lines from immunized LNC and spleen were initially set up in the presence of 25 μg/ml PLP peptide. To generate Th1 lines, cells were cultured in medium containing IL-2 (10 IU/ml) (National Institutes of Health, Bethesda, MD, USA), 10 ng/ml of IL-12 (R&D systems, USA) and 10 μg/ml anti-IL-4 (National Institutes of Health, USA). To generate Th2 lines, cells were cultured in medium containing IL-2 (10 IU/ml) (National Institutes of Health, USA), 10 ng/ml of IL-4 (R&D systems, Minneapolis, Minnesota, USA) and 10 μg/ml of anti-IFNγ (Life Technologies Ltd, Paisley, UK). For Th17 lines, cells were initially cultured in 10 μg/ml anti-IFNγ (Life Technologies Ltd, Paisley, UK), 10 μg/ml anti-IL-4 (National Institutes of Health, USA), 20 ng/ml IL-6 (R&D Systems, USA) and 2 ng/ml TGFβ (R&D Systems, USA) and expanded in medium containing 10 IU/ml IL-2 (National Institutes of Health, USA) and 20 ng/ml IL-23 (R&D Systems, USA). Following the addition of cytokines, cultures were incubated for an additional eight days. Cells were then resuspended in RPMI 1640 medium (Invitrogen, Life Technologies, UK) and 10% FCS, and re-stimulated with 25 to 50 μg/ml peptide in the presence of irradiated, syngeneic splenocytes. The 10-day cycle was repeated as required.

### T cell cytokine assays

T cell proliferation assays of immunized lymph node cells were set up as described above. After 66 h, 50 μl of supernatant was removed from each well to determine cytokine production. The IL-2, IL-4, IL-5, IL-9, IL-10, IL-13, IL-17 and IFNγ content was measured by ELISA (R&D Systems, UK or BD Biosciences, Franklin Lakes, New Jersey, USA). IFNγ (2BScientific Ltd, Upper Heyford, UK), IL-4 (BD Biosciences, USA), IL-17 (R&D Systems, UK). ELISPOT assays (R&D Systems, UK) were performed using 1.2 × 10^4^ T cells and 3 × 10^5^ APCs per well with varying concentrations of peptide. Numbers of spots per well were determined using an AID ELISPOT reader (Autoimmun Diagnostika GmbH, Straßberg, Germany).

### Cytokine storm induction

Mice were immunized intraperitoneally with 200 μg of Staphylococcal Enterotoxin B (SEB) (Sigma Aldrich, UK) or via the footpad with phosphate-buffered saline (PBS) or 50 μg of PLP peptide in CFA. Tail bleed samples were collected prior to immunization and at 2, 24 and 72 hours post immunization. Serum from tail bleed samples was used to measure IFNγ and TNF-α by ELISA (R&D Systems, UK). Thymocytes were harvested at seven days post immunization. PE-anti-CD4 (clone GK1.5, eBioscience, San Diego, California, USA) and fluorescein isothiocyanate (FITC)-anti-CD8 (clone 53 to 6.7, eBioscience, USA) were used to determine the CD4:CD8 ratio of thymocytes and to isolate CD4 single positive thymocytes by cell sorting on a FACS Aria II (BD Biosciences, USA).

### Flow cytometry

Cell suspensions from Th1/Th2 cell lines at eight days post-re-stimulation were labeled with optimal concentrations of the following labeled monoclonals: PE-anti-CD4 (GK1.5), PE-anti-CD40L (MR1), PE-anti-ICOS (7E.17G9), PE-Rat-IgG2b isotype control, FITC-anti-CD4 (GK1.5), FITC-anti-CD69 (H1.2 F3), FITC-anti-CD3 (145-2C11), FITC-anti-CD5 (53–7.3), FITC-anti-CD54 (YN1/1.7.4), FITC-anti-CD127 (A7R34), FITC-Rat IgG2b isotype control; all from eBioscience; FITC-anti-OX40 (OX-86) from Serotec, UK; APC-anti-CD62L (MEL-14) (ImmunoTools, Friesoythe, Germany); and V500-anti-CD4 (RM4-5) (BD Biosciences, USA). Data were collected on FACSCalibur (BD Biosciences, USA) and analyzed with CellQuest software (BD Biosciences, USA) and FlowJo software (Tree Star, Inc., Ashland, Oregon, USA).

### TCR spectratyping

Repertoire analyses were performed using a protocol modified from Pannetier *et al*. [68]. Total RNA was isolated from cell suspensions (Stratagene, Santa Clara, California, USA) followed by cDNA synthesis using SuperScript III (Invitrogen, Life Technologies). For each cDNA, PCR reactions were performed using Vβ primers (Milner *et al*., [64]) and a common 6-carboxyflurorescein-amino-hexy (6-FAM) Cβ primer (6FAM-CTTGGGTGGAGTCACATTTCTC). The PCR products were analyzed on an ABI 3100 Prism Genetic Analyzer (Life Technologies, USA) using Gene Mapper ID Software version 3.2 (Life Technologies, USA).

### TCR subcloning and sequencing

TCRα and TCRβ transcripts were amplified from cDNA prepared from bulk T cell lines (as described in the text) by nested PCR as described [69] and ligated into the pCR2.1 TA cloning vector, following transformation into *E. coli,* individual colonies were sequenced using M13 primers (Cambridge Biosciences, Cambridge, UK) by direct sequencing of purified PCR products. This was carried out using the Big Dye Terminator v1.1 Cycle Sequencing Kit and sequences, analyzed on an Applied Biosystems 3130x1 DNA Analyzer. TCR sequences and CDR3 region lengths were identified according to the International Immunogenetics Information System (IMGT) [70].

### Antigen specific repertoire analysis

Single cells with a CD4^+^CD69^+^ phenotype were sorted for repertoire analysis with a BD FACS Aria II and BD FACSDiva software (BD Biosciences, USA) and RNA extracted from the sorted populations.

### Class II tetramer binding

Tetramer binding was performed in RPMI/FCS and the appropriate concentration of tetramer. H2-Ag^7^ tetramers loaded with PLP56-70 or irrelevant, CLIP103-117 peptide (PVSKMRMATPLLMQA) were used (provided by the NIH Tetramer Facility, Emory University, Atlanta, GA, USA). Cells were incubated with tetramer for 3 h at 37°C before staining with FITC-anti-CD4 (GK1.5, eBioscience, USA) and analyzing by FACS. For experiments with naïve T cells, CD4^+^ T cells were isolated from whole splenocyte cell suspensions by labelling with CD4 (L3T4) Microbeads (Miltenyi Biotec, Bergisch Gladbach, Germany) and positively selecting through an autoMACS™ Separator (Miltenyi Biotec, Germany). For plate-bound tetramer assays, wells of high-binding plates (Corning, Corning, New York, USA) were incubated with 10 μg/ml tetramer overnight. Wells were blocked with 200 μl medium for 1 h at 37°C. A total of 5 × 10^4^ T cells/well and 1 μg/ml of soluble anti-CD28 (clone 37.51, eBioscience, USA) were incubated for 48 h and supernatant collected for ELISA.

### Real-time PCR analysis

RNA samples were prepared using Absolutely RNA® Microprep or Nanoprep spin columns (Stratagene, USA) and cDNA synthesized from 500 ng RNA using SuperScript III reverse transcriptase (Invitrogen, Life Technologies, UK). Real-time PCR reactions were run in triplicate and CT values obtained using a MX3000P real-time PCR machine (Stratagene, USA). Variance in the amount of RNA between samples was controlled for by normalizing to *18S*. Because of differences in amplification efficiencies between primer sets, relative levels of gene expression between samples were calculated by using efficiency curves to convert CT values to numerical values before normalizing each gene of interest value with respect to the *18S* value for the same sample. The sample with the lowest level of gene expression was assigned a value of 1. Levels of gene expression in all other samples were expressed as a value relative to 1. *Gata3, Tbet, IFNγ, Bcl-xl, GAPDH* and *18S* PCR primers, TaqMan MGB probes (FAM dye labeled), as well as TaqMan Universal PCR Master Mix, were purchased from Applied Biosystems (Applied Biosystems-Assays-on-Demand Gene Expression Assay) and *RORγt* primers were purchased from Sigma-Aldrich (sense primer:5′ GTCTGCAAGTCCTTCCGAGAG, antisense primer:5′ ATCTCCCACATTGACTTCCTCTG, FAM labeled probe:5′ [6FAM]CTGCGACTGGAGGACCTTCTACGGC[TAM]).

## Abbreviations

APC: Antigen presenting cell; Bcl2: B-cell lymphoma 2; BcLxL: B-cell lymphoma-extra large; cDNA: Complementary DNA; CDR3: Complementarity determining region 3; CFA: Complete Freund’s adjuvant; CTLA-4: Cytotoxic T-lymphocyte antigen 4; DC: Dendritic cell; EAE: Experimental autoimmune encephalomyelitis; ELISA: Enzyme-linked immunosorbent assay; FACS: Fluorescence activated cell sorter; FITC: Fluorescein isothiocyanate; ICAM-1: Intercellular adhesion molecule-1; ICOS: Inducible T-cell co-stimulator; IFA: Incomplete Freund’s adjuvant; IFNγ: Interferon gamma; IL-2: 4, 9, 10, 12, 13, 17, 18, 23, Interleukin-2, 4, 9, 10, 12, 13, 17, 18, 23; LCMV: Lymphochoriomeningitis virus; LNC: Lymph node cells; MHC: Major histocompatibility complex; NOD.E: Non-obese diabetic, H2-E transgenic; PCR: Polymerase chain reaction; PLP: Proteolipoprotein; pMHC: peptide/major histocompatibility complex; SEA: Schistosome egg antigen; SEB: Staphylococcal enterotoxin B; TCR: T cell receptor; TGFβ: Transforming growth factor beta; Th1: 2, 17, T helper 1, 2, 17; TLR: Toll-like receptor; TNFα: Tumor necrosis factor alpha.

## Competing interests

The authors declare that they have no competing interests.

## Authors’ contributions

CR acquired the data, analyzed and interpreted the data, and Drafted or revised the manuscript. DC, ER, KQ, DK and JL-H acquired the data. DA analyzed and interpreted the data, and drafted or revised the manuscript. RB was responsible for conception and design of the study, acquisition of data, analysis and interpretation of data, and drafting or revising the manuscript. All authors read and approved the final manuscript.

## Authors’ information

CR and DC are post-doctoral research associates. ER and KQ are PhD students. DK is a MSc student. JLH is a DNA sequencing Facility Manager. DA is a Professor of Immunology. RB is the Principal Investigator and Head of the Lung Immunology Group.

## Supplementary Material

Additional file 1 **Intracellular cytokine staining for antigen specific T cell lines.** A representative example of intracellular cytokine staining for antigen specific T cell lines grown in **(A)** Th1 (n = 6) and **(B)** Th17 (n = 6) culture media. Cell lines were grown through one re-stimulation in polarizing cell culture medium before intracellular cytokine staining with FITC-conjugated IL-17 and PE-conjugated IFNγ antibodies. Note that IFNγ producing cells readily differentiate within Th17 cultures, notwithstanding clear-cut overall differences in preferential TCR usage (Tables 1 and 3) and binding avidity (Figures 6 and 7). Click here for file

Additional file 2 **No bias in the TCR β chain repertoire of naïve TCRVα chain transgenics at baseline or in a primary response in a DLN at Day 10 post-immunization as demonstrated by spectratype analysis.** TCR β chain repertoire by spectratype analysis of **(A)** naïve TCRα transgenic and littermate control splenocytes and **(B)** primed DLNs at Day 10 post immunization with PLP 56 to 70 in CFA. V region specific primers were used in combination with a FAM labeled constant region primer to amplify TCRβ chain sequences from T cell cDNA templates. Data shown are representative of experiments carried out with 10 TCRα transgenic and 10 littermate controls and three independently performed experiments. Click here for file

Additional file 3 **No difference in T-bet transcription between TCRαβ transgenic and littermate control cell lines.** TCRαβ transgenic cell lines (black bars) (n = 3) and littermate control lines (white bars) (n = 5) were established from primed DLN cells from mice primed 10 days earlier with PLP56 to 70/CFA and re-stimulated every 10 days through to four cycles in the absence of exogenous polarization. At each re-stimulation the relative expression of *T-bet* was determined. Error bars indicate SE. Click here for file

Additional file 4: Figure S5 TCRαβ transgenics show strong functional T cell activation and absence of an enhanced apoptotic program. TCRVαβ transgenic (n = 4) and littermate control (n = 5) mice were primed with PLP56 to 70 on Day 0 (footpad, CFA) and Day 28 (flank, IFA). DLN and splenocytes were harvested at Day 10, Day 28 and Day 32. At the Day 32, CD4^+^ T cells were analyzed for expression of **(A)** the pro-survival factor *Bclxl* by real time PCR (Day 32) and **(B)** CD127 (Day 28), and **(C)** CD62L (Day 28) by flow cytometry. Statistical significance between groups was determined using an unpaired t test. Error bars indicate SE. Click here for file

Additional file 5 **Peptide priming of TCRVα, TCRVαβ transgenics or littermate controls does not result in a systemic cytokine storm or reduced thymocyte numbers. (A)** Littermate controls, TCRVα and TCRVαβ transgencis were immunized with 200 μg SEB (striped bars) (littermate controls, n = 5; TCRVα, n = 9; TCRVαβ, n = 9), PBS/CFA (white bars) (littermate controls, n = 4; TCRVα, n = 4; TCRVαβ, n = 4), or 50 μg PLP/CFA (black bars) (littermate controls, n = 4; TCRVα, n = 4; TCRVαβ, n = 4). **(B)** Serum samples were collected at time points 0, 2, 24 and 72 hours from mice injected with SEB (striped bars), PBS/CFA (white bars) or 50 μg PLP/CFA (black bars) and IFNγ (top row) and TNF-α (middle row) levels measured by ELISA. On Day 7, total thymocyte counts and CD4/CD8 thymocyte ratios were determined (bottom row). CD4 single positive thymocytes were isolated by cell sorting and the CDR3β repertoire of **(C)** littermate controls and **(D)** TCRVα transgenic mice immunized with PLP/CFA determined by TCR subcloning and sequencing. Click here for file
