# Supplementary figures and images for: Elongated TCR alpha chain CDR3 favors an altered CD4 cytokine profile
Source: BMC Biol. 2014 May 9;12:32. doi: 10.1186/1741-7007-12-32 (PMC4046507; doi:10.1186/1741-7007-12-32)

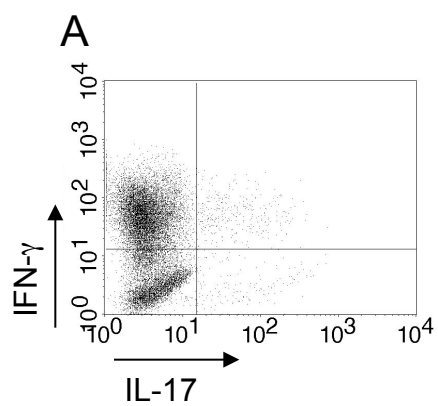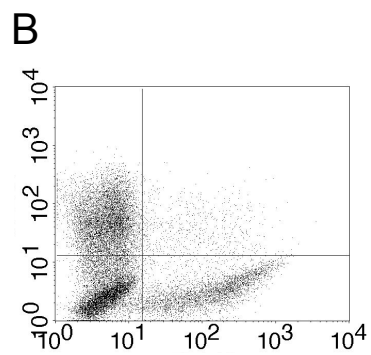

Supplement: Additional file 1 — Intracellular cytokine staining for antigen specific T cell lines. A representative example of intracellular cytokine staining for antigen specific T cell lines grown in (A) Th1 (n = 6) and (B) Th17 (n = 6) culture media. Cell lines were grown through one re-stimulation in polarizing cell culture medium before intracellular cytokine staining with FITC-conjugated IL-17 and PE-conjugated IFNγ antibodies. Note that IFNγ producing cells readily differentiate within Th17 cultures, notwithstanding clear-cut overall differences in preferential TCR usage (Tables 1 and 3) and binding avidity (Figures 6 and 7). [file 1741-7007-12-32-S1.pdf]

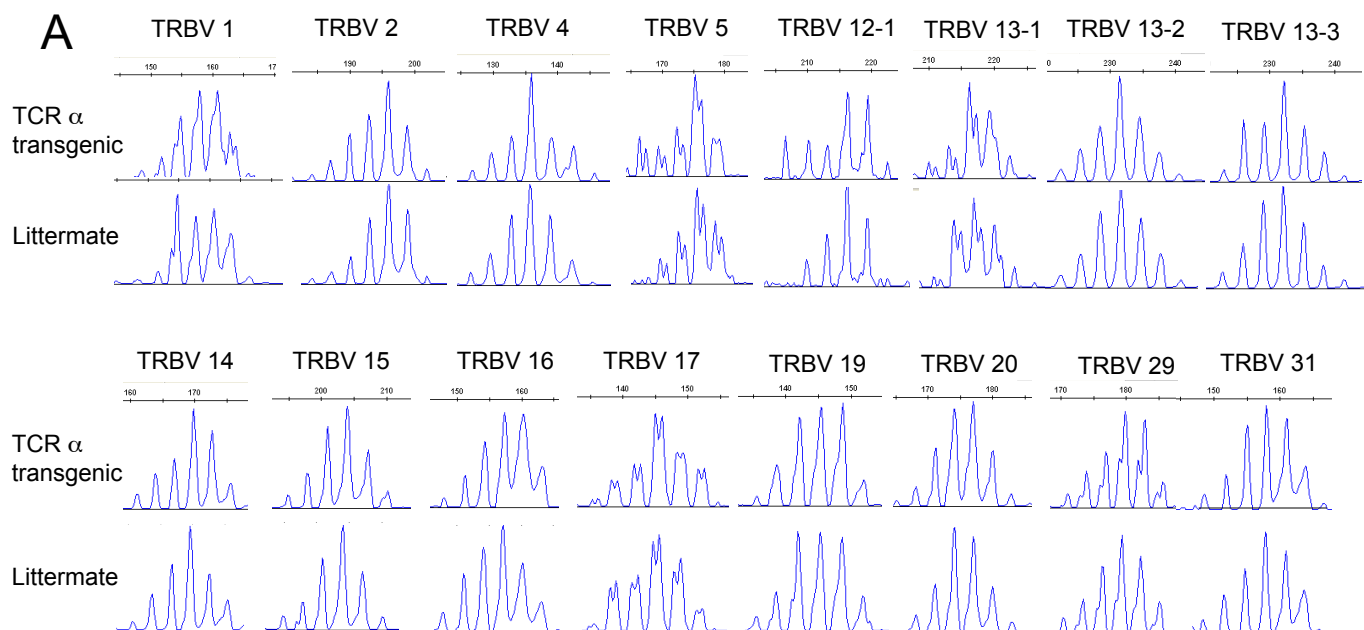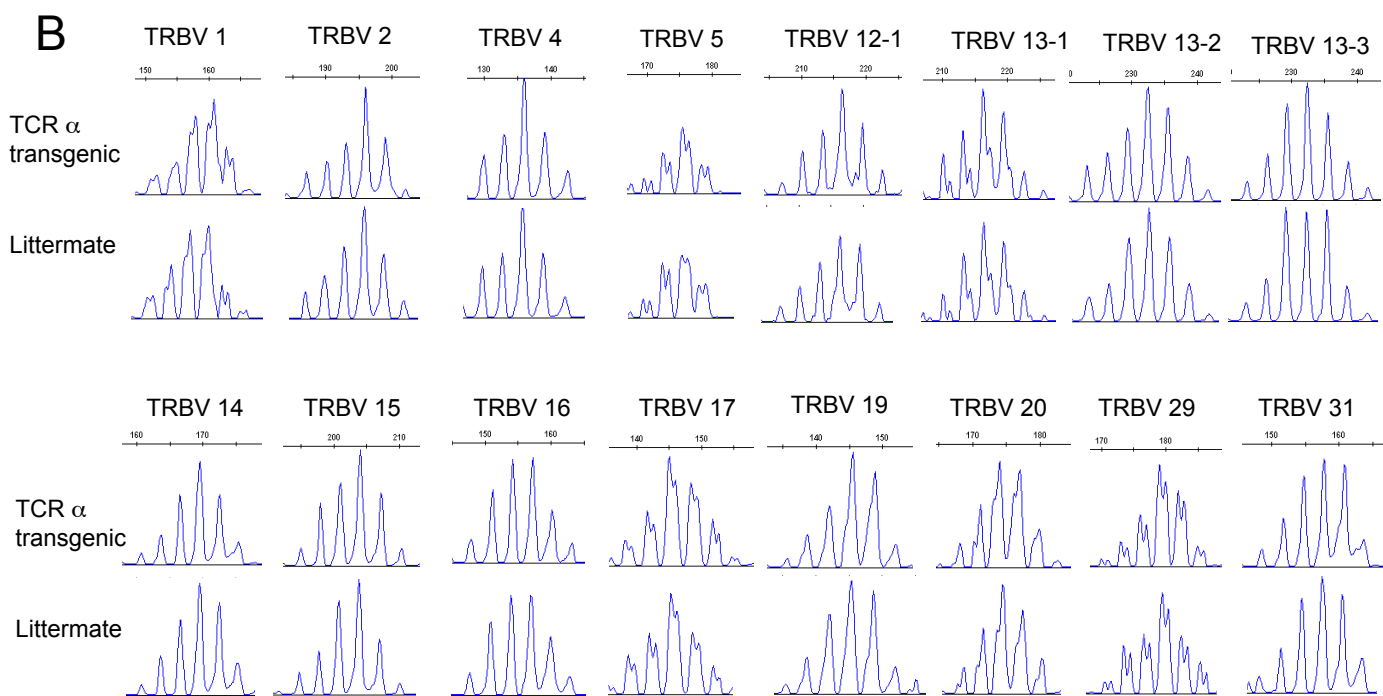

Supplement: Additional file 2 — No bias in the TCR β chain repertoire of naïve TCRVα chain transgenics at baseline or in a primary response in a DLN at Day 10 post-immunization as demonstrated by spectratype analysis. TCR β chain repertoire by spectratype analysis of (A) naïve TCRα transgenic and littermate control splenocytes and (B) primed DLNs at Day 10 post immunization with PLP 56 to 70 in CFA. V region specific primers were used in combination with a FAM labeled constant region primer to amplify TCRβ chain sequences from T cell cDNA templates. Data shown are representative of experiments carried out with 10 TCRα transgenic and 10 littermate controls and three independently performed experiments. [file 1741-7007-12-32-S2.pdf]

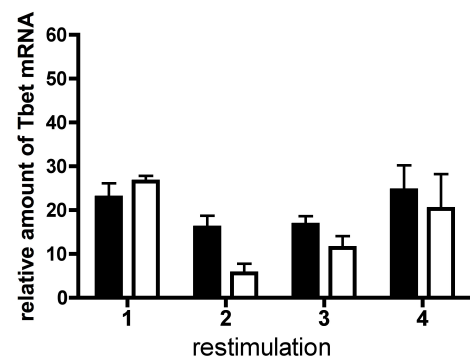

Supplement: Additional file 3 — No difference in T-bet transcription between TCRαβ transgenic and littermate control cell lines. TCRαβ transgenic cell lines (black bars) (n = 3) and littermate control lines (white bars) (n = 5) were established from primed DLN cells from mice primed 10 days earlier with PLP56 to 70/CFA and re-stimulated every 10 days through to four cycles in the absence of exogenous polarization. At each re-stimulation the relative expression of T-bet was determined. Error bars indicate SE. [file 1741-7007-12-32-S3.pdf]

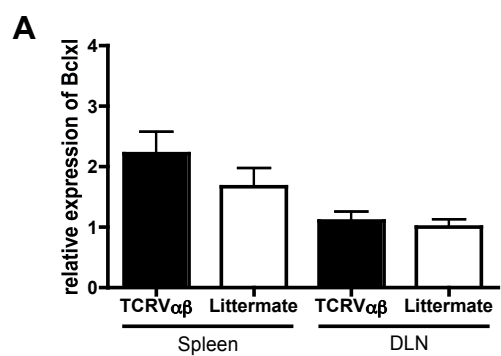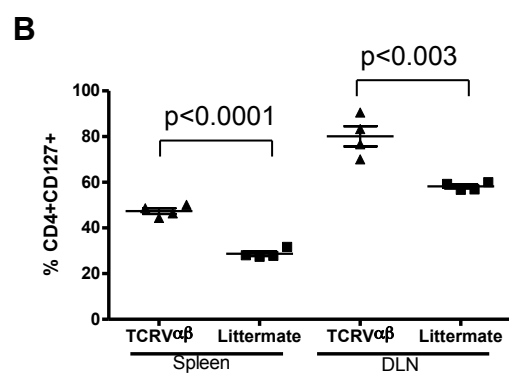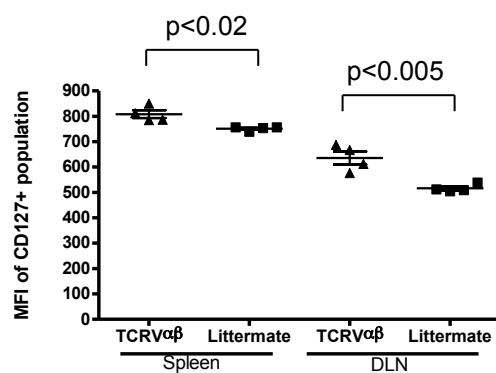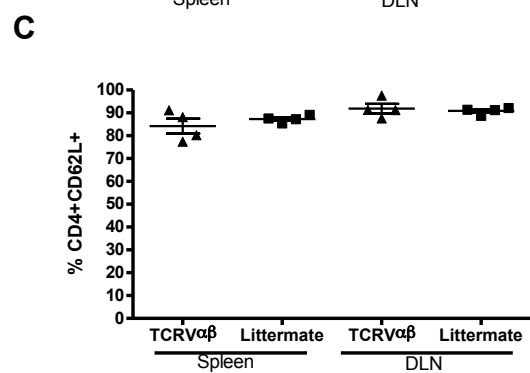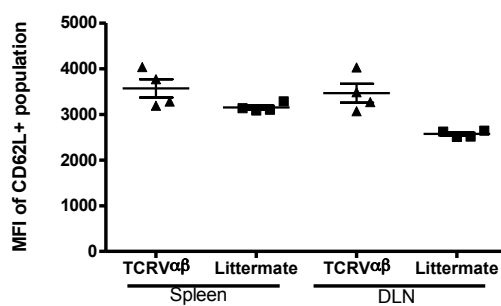

Supplement: Additional file 4: Figure S5 — TCRαβ transgenics show strong functional T cell activation and absence of an enhanced apoptotic program. TCRVαβ transgenic (n = 4) and littermate control (n = 5) mice were primed with PLP56 to 70 on Day 0 (footpad, CFA) and Day 28 (flank, IFA). DLN and splenocytes were harvested at Day 10, Day 28 and Day 32. At the Day 32, CD4+ T cells were analyzed for expression of (A) the pro-survival factor Bclxl by real time PCR (Day 32) and (B) CD127 (Day 28), and (C) CD62L (Day 28) by flow cytometry. Statistical significance between groups was determined using an unpaired t test. Error bars indicate SE. [file 1741-7007-12-32-S4.pdf]
