# Supplementary material for: Elongated TCR alpha chain CDR3 favors an altered CD4 cytokine profile
Source: BMC Biol. 2014 May 9;12:32. doi: 10.1186/1741-7007-12-32 (PMC4046507; doi:10.1186/1741-7007-12-32)
Supplement: Additional file 5 — Peptide priming of TCRVα, TCRVαβ transgenics or littermate controls does not result in a systemic cytokine storm or reduced thymocyte numbers. (A) Littermate controls, TCRVα and TCRVαβ transgencis were immunized with 200 μg SEB (striped bars) (littermate controls, n = 5; TCRVα, n = 9; TCRVαβ, n = 9), PBS/CFA (white bars) (littermate controls, n = 4; TCRVα, n = 4; TCRVαβ, n = 4), or 50 μg PLP/CFA (black bars) (littermate controls, n = 4; TCRVα, n = 4; TCRVαβ, n = 4). (B) Serum samples were collected at time points 0, 2, 24 and 72 hours from mice injected with SEB (striped bars), PBS/CFA (white bars) or 50 μg PLP/CFA (black bars) and IFNγ (top row) and TNF-α (middle row) levels measured by ELISA. On Day 7, total thymocyte counts and CD4/CD8 thymocyte ratios were determined (bottom row). CD4 single positive thymocytes were isolated by cell sorting and the CDR3β repertoire of (C) littermate controls and (D) TCRVα transgenic mice immunized with PLP/CFA determined by TCR subcloning and sequencing. [file 1741-7007-12-32-S5.pdf]

A

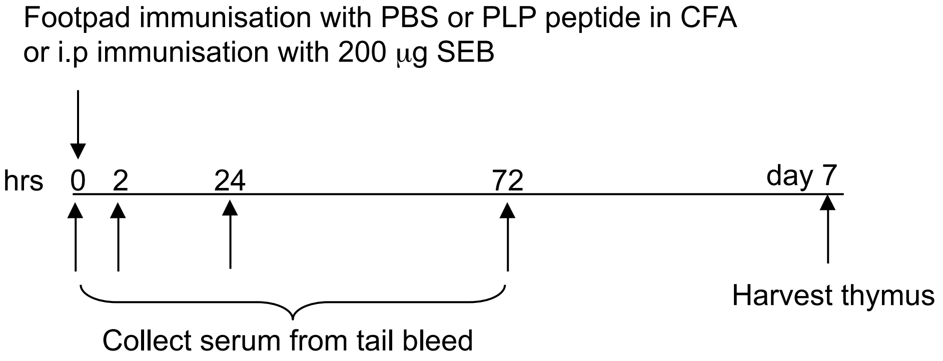

Footpad immunisation with PBS or PLP peptide in CFA
or i.p immunisation with 200 μg SEB
hrs
0
2
24
72
day 7
Collect serum from tail bleed
Harvest thymus

B

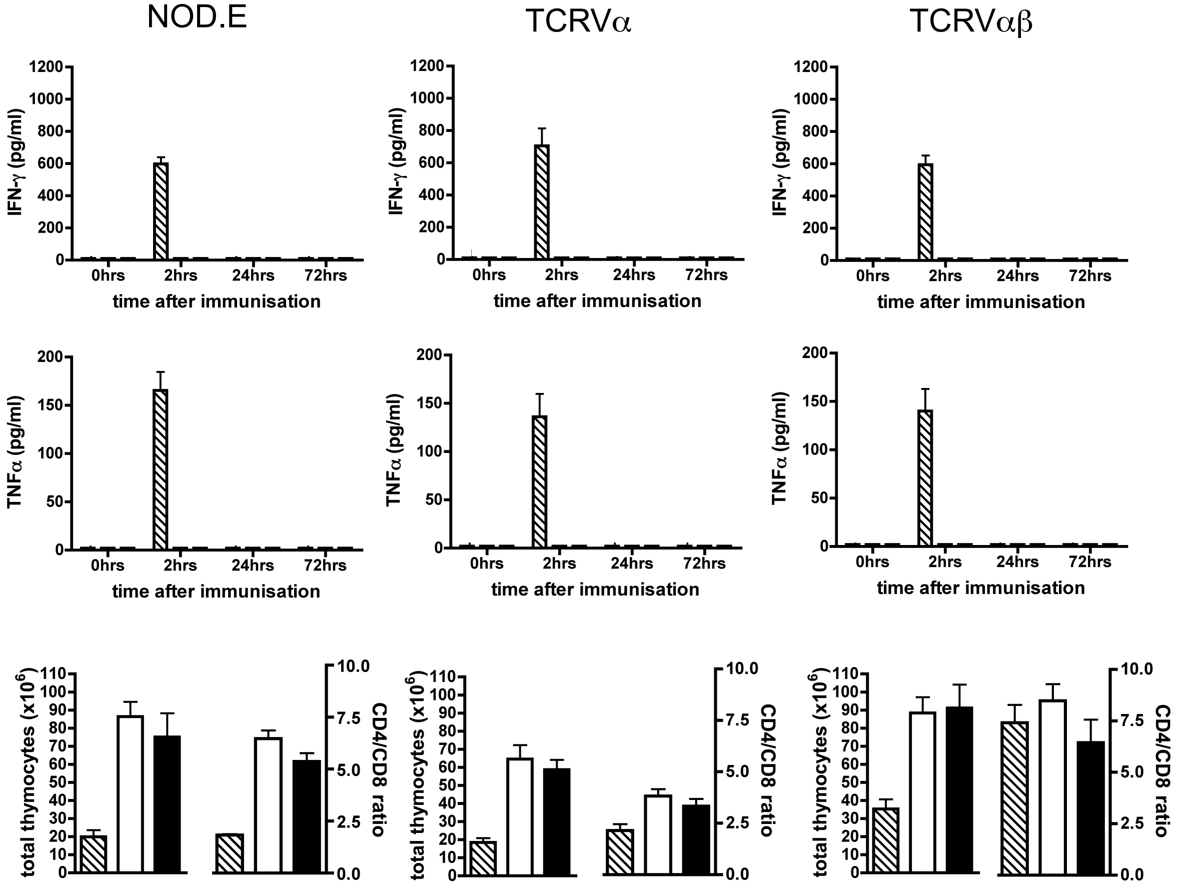

NOD.E
TCRVα
TCRVαβ
IFN-γ (pg/ml)
0
200
400
600
800
1000
1200
0hrs
2hrs
24hrs
72hrs
time after immunisation
TNFα (pg/ml)
0
50
100
150
200
total thymocytes (x10^6)
0
10
20
30
40
50
60
70
80
90
100
110
CD4/CD8 ratio
0.0
2.5
5.0
7.5
10.0

C

	CDR3 region		CDR3 length	%
QDSAVYLC	ASLVPGGGYAEQFF	FGPG	14	10.1
DDSATYFC	ASSQERQGVSNERLF	FGHG	15	5.8
EDRGLYLC	GAREQGDSDYT	FGSG	11	5.8
SHSGFYLC	AWSLFSAETLY	FGSG	11	5.8
NQTSVYFC	ASSLGTGGDEQY	FGPG	12	5.8
DDSATYFC	ASSQSDANTEVF	FGKG	12	2.9
DDSATYFC	ASSHGGDAETLY	FGSG	12	2.9
DDSATYFC	ASSQEGDTNNERLF	FGHG	14	2.9
DDSATYFC	ASSQGTGGGAETLY	FGSG	14	2.9
DDSATYFC	ASSHPGQGNYAEQF	FGPG	14	2.9
SQTSVYFC	ASGDRGRLGGQDTQY	FGPG	15	2.9
SQTSLYFC	ASSEGLGDNYAEQF	FGPG	14	2.9
SQTSLYFC	ASTWGDSYEQYF	FGPG	12	2.9
SQTSLYFC	ASSDAPNSDYT	FGSG	11	2.9
SQTSLYFC	ASKTGNTGQLY	FGEG	11	2.9
SQTSVYFC	ASGDAGAEEVF	FGKG	11	2.9
SQTSVYSC	ASGDAREYAEQF	FGPG	12	2.9
SQTSVYFC	ASGDFRDRGTTEVF	FGKG	14	2.9
EDRGLYLC	GASPGTSNTEVF	FGKG	12	2.9
QDSAVYLC	ASSLGNQDTQY	FGPG	11	2.9
SHSGFYLC	AWSPGTGGYEQY	FGPG	12	2.9
EDSAVYFC	ASSQGTGSAETLY	FGSG	13	2.9
EDSAVYFC	ASSPYRGQDTQY	FGPG	12	2.9
EDSAVYLC	ASSLTGGRYAEQF	FGPG	13	2.9
EDSAVYLC	ASSHRGSNTEVF	FGKG	12	2.9
EDSAVYLC	ASSYGVSNERLF	FGHG	12	2.9
NQTSVYFC	ASGPTGGNYAEQF	FGPG	13	2.9
EDSAVYLC	ASSYGVSNERLF	FGHG	12	2.9

	CDR3 region		CDR3 length	%
QDSAVYLC	ASSLVGGAETLY	FGSG	12	6.5
EDRGLYLC	GARDGGSYEQY	FGPG	11	6.5
QDSAVYLC	ASSSGPNERLF	FGHG	11	3.0
QDSAVYLC	ASSLQGGYQDTQY	FGPG	13	3.0
QDSAVYLC	ASSLGNQDTQY	FGPG	10	3.0
SQTSVYFC	ASGDPRGGGNTLY	FGEG	13	3.0
SQTSLYFC	ASSQGVYEQY	FGPG	10	3.0
SQTSLYFC	ASSHSSAETLY	FGSG	11	3.0
SQTSVYFC	ASGDAGTGGRY	FGPG	10	3.0
SQTSLYFC	ASNPGGSSYEQY	FGPG	12	3.0
SQTSVYFC	ASGGANRVSQNTLY	FGAG	14	3.0
DDSATYFC	ASSQDDNAETLY	FGSG	12	3.0
DDSATYFC	ASSQDLGQDAQY	FGPG	12	3.0
DDSATYFC	ASSQDWGSQNTLY	FGAG	12	3.0
DDSATYFC	ASSQGQGNERLF	FGHG	11	3.0
DDSATYFC	ASSQDWWGSSYEQY	FGPG	13	3.0
DDSATYFC	ASSQVGIQDTQY	FGPG	11	3.0
DDSATYFC	ASSQLGKDTQY	FGPG	10	3.0
DDSATYFC	ASSQEPSQDTQY	FGPG	11	3.0
NEMAVFLC	ASSITGEGSPLY	FAAG	12	3.0
NEMAVFLC	ASSRDRVRERLF	FGHG	12	3.0
NEMAVFLC	ASSISNTGQLY	FGEG	10	3.0
EDSAVYFC	ASSQDWGGDAEQF	FGPG	13	3.0
EDSAVYLC	ASSLGTKNTLY	FGAG	10	3.0
SHSGFYLC	AWRGLGTSQNTLY	FGAG	13	3.0
SHSGFYLC	AWSLLGGIDEQY	FGPG	11	3.0
EYSAMYLC	ASSRDWDYEQY	FGPG	11	3.0
QDSSVYSC	ASSRNERLF	FGHG	9	3.0
GDSSIYFC	SSSEGLGGANTGQLY	FGEG	14	3.0
PQTSVYFC	ASGEARDNNERLF	FGHG	12	3.0
KDSAVYLC	ASSLGSQNTLY	FGAG	10	3.0

	CDR3 region		CDR3 length	%
DDSATYFC	ASSQEQANYAEQF	FGPG	13	6.6
EDSAVYLC	ASSYWKDTQY	FGPG	10	6.6
DDSATYFC	ASSQDWGDAEQF	FGPG	12	3.3
DDSATYFC	ASSQLGKDTQY	FGPG	11	3.3
DDSATYFC	ASSQRDWQDTQY	FGPG	12	3.3
DDSATYFC	ASSQDLDSSQNTLY	FGAG	14	3.3
DDSATYFC	ASSQDPQKYEQY	FGPG	12	3.3
DDSATYFC	ASSQLGKDTQY	FGPG	11	3.3
DDSATYFC	ASSQDEAAEQF	FGPG	11	3.3
EDRGLYLC	GARDGDTQY	FGPG	9	3.3
EDSAVYLC	ASSLGTKNTLY	FGAG	11	3.3
SQTSLYFC	ASSDPGNQDTQY	FGPG	12	3.3
SQTSLYFC	ASNRDRATEVF	FGKG	11	3.3
SQTSLYFC	ASRPGQDTEVF	FGKG	11	3.3
SQTSLYFC	ASSETRDRDNSPLY	FAAG	14	3.3
SQTSLYFC	ASSAGTGQLY	FGEG	10	3.3
SQTSLYFC	ASSDAGWGDDEQY	FGPG	13	3.3
NEMAVFLC	ASSIGNGYSGNTLY	FGEG	14	3.3
NEMAVFLC	ASSISNTGQLY	FGEG	11	3.3
NEMAVFLC	ASSTGEGAEQF	FGPG	11	3.3
NEMAVFLC	ASSWSGGYAEQF	FGPG	12	3.3
SQTPVYFC	ASGDVNTLY	FGAG	9	3.3
SQTSLYFC	ASSETRDRDNSPLY	FAAG	14	3.3
QDSAVYLC	ASSLQGGYQDTQY	FGPG	13	3.3
EDSAVYLC	ASSLGTKNTLY	FGAG	11	3.3
SHSGFYLC	AWSRDWGGAGTGQLY	FGEG	15	3.3
DDSATYFC	ASSQDYWDEQY	FGPG	11	3.3
EYSAMYLC	ASSTRQGANERLF	FGHG	13	3.3

	CDR3 region		CDR3 length	%
QDSAVYLC	ASSSQGISNERLF	FGHG	13	6.6
SQTSLYFC	ASSDVSGTTNTGQLY	FGEG	15	6.6
SHSGFYLC	AWSLYWGGGYEQY	FGPG	13	6.6
EDSAVYFC	ASSPGQFSNERLF	FGHG	13	6.6
QDSAVYLC	ASSLGLGDEQYF	FGPG	12	3.2
QDSAVYLC	ASSSLRTDQDTQY	FGPG	13	3.2
SQTSVYFC	ASGEDRDWGYAEQF	FGPG	14	3.2
SQTSLYFC	ASSDLAGGYAEQF	FGPG	13	3.2
SQTSLYFC	ASSGSLYEQY	FGPG	10	3.2
SQTSLYFC	ASSDRLGGSTGQLY	FGEG	14	3.2
SQTSLYFC	ASRTGGAGDTQY	FGPG	12	3.2
SQTSVYFC	ASGDRGNERLF	FGHG	11	3.2
SQTSLYFC	ASSPPGQNTLY	FGAG	11	3.2
SQTSLYFC	ASSDAGDTGQLY	FGEG	12	3.2
SQTSLYFC	ASSDPGTYYAEQF	FGPG	13	3.2
SQTSVYFC	ASGDAGGNQDTQY	FGPG	13	3.2
DDSATYFC	ASSRDNSYEQY	FGPG	11	3.2
DDSATYFC	ASSQQVAEVF	FGKG	10	3.2
DDSATYFC	ASSQDRGGTKRLF	FGHG	13	3.2
DDSATYFC	ASSQNRDWGDEQF	FGPG	13	3.2
DDSATYFC	ASSQRQSGAETLY	FGSG	13	3.2
DDSATYFC	ASSQDPLNTLY	FGAG	11	3.2
EDSAVYLC	ASSRLGASAETLYF	FGSG	14	3.2
EDSAVYFC	ASSRDSVSYEQY	FGPG	12	3.2
EDSAVYFC	VSSQDKAGANTEVF	FGKG	14	3.2
EDSAVYLC	ASSFRDWENAETLY	FGSG	14	3.2
SHSGFYLC	AWKDWGDEQY	FGPG	10	3.2

Mean CDR3β length 12.10 ± 0.13 (SE) (n = 128)

D

	CDR3 region		CDR3 length	%
SQTSLYFC	ASSDAWGGQDTQY	FGPG	13	8.0
NEMAVFLC	ASSQDWAGQLY	FGEG	11	8.0
DDSATYFC	ASSPDWGNTLY	FGAG	11	3.5
DDSATYFC	ASSQDSTGGDDTQY	FGPG	14	3.5
DDSATYFC	ASSQSRGNSPLY	FAAG	12	3.5
DDSATYFC	ASSQVGIQDTQY	FGPG	12	3.5
DDSATYFC	ASSQEGGASAETLY	FGSG	14	3.5
SQTSLYFC	ASSGTGNNQAPL	FGEG	12	3.5
SQTSLYFC	ASRADSAETLY	FGSG	11	3.5
SQTSLYFC	ASSDGGQNTLY	FGAG	11	3.5
SQTSVYFC	ASGDAAGNTERLF	FGHG	13	3.5
SQTSVYFC	ASGDNSGNTLY	FGEG	11	3.5
SQTSLYFC	ASSAGLGVSYEQY	FGPG	13	3.5
SQTSLYFC	ASSVTDSGNTLY	FGEG	12	3.5
NEMAVFLC	ASSTGEGAEQF	FGPG	11	3.5
NEMAVFLC	ASSRDRVRERLF	FGHG	12	3.5
NEMAVFLC	ASSIGNGYSGNTLY	FGEG	14	3.5
NEMAVFLC	ATGTGVSEQY	FGPG	10	3.5
NEMAVFLC	ASSMIGGGSYEQY	FGPG	13	3.5
NEMAVFLC	ASSITGEGSPLY	FAAG	12	3.5
QDSAVYLC	ASSSGPNERLF	FGHG	11	3.5
QDSAVYLC	ASSFRDRHNERLF	FGHG	13	3.5
QDSAVYLC	ASSSGQNERLF	FGHG	11	3.5
QDSAVYLC	ASSLGTAGNTLY	FGEG	12	3.5
EYSAMYLC	ASSGLGANTGQLY	FGEG	13	3.5
EDSAVYLC	ASSYSGGEQY	FGPG	10	3.5

	CDR3 region		CDR3 length	%
EYSAMYLC	ASSGLGANTGQLY	FGEG	13	7.6
NQTSVYFC	ASDRASDYT	FGSG	9	7.6
EDRGLYLC	GALRDRGNERLF	FGHG	12	7.6
DDSATYFC	ASSQDLLSSAETLYF	FGSG	15	3.5
DDSATYFC	ASSQDLGGRYAEQF	FGPG	14	3.5
DDSATYFC	ASSRDWGYEQY	FGPG	11	3.5
DDSATYFC	ASSQDRGGTKDLF	FGHG	13	3.5
DDSATYFC	ASSQGQYEQY	FGPG	10	3.5
DDSATYFC	ASSQDQGANSDYT	FGSG	13	3.5
DDSATYFC	ASSRGQTYEQY	FGPG	11	3.5
SQTSVYFC	ASGDAANSDYT	FGSG	11	3.5
SQTSLYFC	ASSPPGQNTLYF	FGAG	12	3.5
SQTSLYFC	ASRGLGQDTQYF	FGPG	12	3.5
SQTSVYFC	ASGYEQY	FGPG	7	3.5
SQTSLYFC	ASSGTGGYEQY	FGPG	11	3.5
SQTSLYFC	ASSDRLGGSTGQLY	FGEG	14	3.5
EYSAMYLC	ASSGLGGANTGQLY	FGEG	14	3.5
EYSAMYLC	ASSSGGTGNTLYF	FGEG	13	3.5
QDSAVYLC	ASSSGQNERLF	FGHG	11	3.5
QDSAVYLC	ASSRDNYEQY	FGPG	10	3.5
QDSAVYLC	ASSLGLGDEQY	FGPG	11	3.5
QDSAVYLC	ASSSQGISNERLF	FGHG	13	3.5
SQTSLYFC	ASSLTDSAETLY	FGSG	12	3.5
SQTSVYFC	ASGEGDWGYEQYF	FGPG	13	3.5
EDSAVYFC	ASSQRQTGQLY	FGEG	11	3.5

	CDR3 region		CDR3 length	%
EDRGLYLC	GASPGTSNTEVF	FGKG	12	13.6
EDSAVYLC	ASSQVGGAGQY	FGPG	11	9.6
EDRGLYLC	GAREQGDSDYT	FGSG	11	9.6
DDSATYFC	ASSHPGQGNYAEQF	FGPG	14	9.6
EDSAVYFC	ASSQEGDWGYEQY	FGPG	13	4.8
DDSATYFC	ASSQDADAEQF	FGPG	11	4.8
DDSATYFC	ASSQDGGGSNYAEQF	FGPG	15	4.8
DDSATYFC	ASSQGTGGGAETLY	FGSG	14	4.8
DDSATYFC	ASSQEKYAEQF	FGPG	11	4.8
DDSATYFC	ASSQEGGANTEVF	FGKG	13	4.8
SQTSVYFC	ASGDWSYEQY	FGPG	10	4.8
SQTSLYFC	ASSDRVSNERL	FGHG	11	4.8
SHSGFYLC	AWSLFSAETLY	FGSG	11	4.8
QDSAVYLC	ASLVPGGGYAEQF	FGPG	13	4.8
GLSGVSLC	KQLRWTGGNYAEQF	FGPG	14	4.8
EDSAVYFC	ASSPPGQNTEVF	FGKG	12	4.8

	CDR3 region		CDR3 length	%
EYSAMYLC	ASSGLGANTGQLY	FGEG	13	44.0
EDKGLNLC	GVLRDRGNERLF	FGHG	12	7.0
DDSATYFC	ASSQGQYEQY	FGPG	10	7.0
EDSAVYFC	ASSLGAQDTQY	FGPG	11	3.5
EDSAVYFC	ASSQGTGDEQY	FGPG	11	3.5
QDSAVYLC	ASSSGQNERLF	FGHG	11	3.5
QDSAVYLC	ASSLDNTEVF	FGKG	10	3.5
SQTSVYFC	ASGDAEGLGGLEQF	FGPG	14	3.5
SQTSVYFC	ASGGGFLNSPLY	FAAG	12	3.5
EYSAMYLC	ASSPRDRGLGNTLY	FGEG	14	3.5
EDSDLNLC	ASSQGTGGEQ	FGPG	10	3.5
EDRGLYLC	GALRDRGNERLF	FGHG	12	3.5
SQTSLYFC	ASRDRGNSDYTF	FGSG	12	3.5
LMTRPYFC	AHQIWGALLT	FGAG	10	3.5
EDSAVYLC	ASSLSSGVYEQY	FGPG	12	3.5

Mean CDR3β length 12.02 ± 0.14 (SE) (n = 105)
